# Boletaceae in China: Taxonomy and phylogeny reveal a new genus, two new species, and a new record

**DOI:** 10.3389/fmicb.2022.1052948

**Published:** 2023-02-02

**Authors:** Yang Wang, Li-Ying Wang, Dan Dai, Zheng-Xiang Qi, Zhen-Hao Zhang, Ya-Jie Liu, Jia-Jun Hu, Peng Zhang, Yu Li, Bo Zhang

**Affiliations:** ^1^Joint International Research Laboratory of Modern Agricultural Technology, Ministry of Education, Jilin Agricultural University, Changchun, China; ^2^College of Plant Protection, Shenyang Agricultural University, Shenyang, China; ^3^Engineering Research Center of Chinese Ministry of Education for Edible and Medicinal Fungi, College of Plant Protection, Jilin Agricultural University, Changchun, China; ^4^Institute of Agricultural Applied Microbiology, Jiangxi Academy of Agricultural Sciences, Nanchang, China; ^5^Mudanjiang Sub-Academy, Heilongjiang Academy of Agricultural Sciences, Mudanjiang, Heilongjiang, China

**Keywords:** Boletales, new taxa, *Hemilanmaoa*, *Lanmaoa*, *Phylloporus*, *Porphyrellus*

## Abstract

Boletaceae, the largest family in Boletales, has been attracted by mycologists in the world due to its diverse morphology and complex history of evolution. Although considerable work has been done in the past decades, novel taxa are continually described. The current study aimed to introduce three new taxa and one new record of Boletaceae from China. The morphological descriptions, color photographs, phylogenetic trees to show the positions of the taxa, and comparisons with allied taxa are provided. The new genus *Hemilanmaoa* is unique in the *Pulveroboletus* group, and *Hemilanmaoa retistipitatus* was introduced as the type species. It can be distinguished by its bluing basidioma when injured, a decurrent hymenophore, a stipe covered with distinct reticulations, and a fertile stipitipellis. *Porphyrellus pseudocyaneotinctus* is characterized by its pileipellis consisting of broadly concatenated cells and thin-walled caulocystidia in *Porphyrellus*. In *Phylloporus*, *Phylloporus biyangensis* can be distinguished by its hymenophores that change to blue when injured and yellow basal mycelium. *Lanmaoa angustispora*, as a new record, is first reported in Northern China. Internal transcribed spacer (ITS), 28S rDNA (28S), translation elongation factor 1-alpha (tef1-α), RNA polymerase II subunit 1 (rpb1), and RNA polymerase II subunit 2 (rpb2) were employed to execute phylogenetic analyses.

## Introduction

The symbiotic systems between fungi and plants were recognized to accelerate the process of vascular plants invading land (Treub, 1884; Pirozynski and Malloch, 1975; Malloch et al., 1980). To date, there are four different mycorrhizal association types, *viz*. vesicular-arbuscular mycorrhizas (VAM), ectomycorrhizas (ECM), orchid mycorrhizas, and ectendo-, arbutoid, and monotropoid associations (Brundrett et al., 1996). The ability that ectomycorrhizal fungi symbiosis with the host can enhance the utilization efficiency of soil nutrition in plants and their ability to resist pests, which consequently improves the survival rates of plants in the ecosystem (Marx, 1972; Trappe, 1977). Meanwhile, competing with saprotrophic fungi in carbon recycling and other nutrients can dramatically influence the balance of forest ecosystems (Tedersoo et al., 2010; Wu et al., 2022). As a species-rich family in the order Boletales E.-J. Gilbert, a majority of species in Boletaceae Chevall. are ectomycorrhizal fungi. Mushrooms in the Boletaceae are characterized by their large, fleshy, and usually brilliantly colored basidioma and hymenophore that are tubulose, lamellate, or loculus.

Over the last few decades, the rapid development of molecular techniques has immensely improved the resolution of the fungal tree of life (He et al., 2019; Naranjo-Ortiz and Gabaldón, 2019; Li et al., 2021). The modern phylogenetic analyses are essential to reassess and resolve the traditional taxonomy of Boletaceae (Skrede et al., 2011; Yang, 2011; Halling et al., 2012b,^2015^; Nuhn et al., 2013; Arora and Frank, 2014; Wu et al., 2014; Orihara et al., 2016; Kuo and Ortiz-Santana, 2020). In the course of DNA-based research, many new genera were proposed within Boletaceae, and intra-relationships of many complex groups tend to be clarified (Yang et al., 2003; Dentinger et al., 2010; Halling et al., 2012b,^2015^; Arora and Frank, 2014; Wu et al., 2014, 2021; Cui et al., 2016; Orihara et al., 2016; Frank et al., 2020; Li and Yang, 2021; Biketova et al., 2022). Based on materials collected from China, Chinese mycologists have made huge contributions to the modern taxonomical system of Boletaceae—especially some fantastic studies were done by Wu et al. (2014) and Wu et al., 2016a,b. In 2014, Wu et al. (2014) redefined seven major clades within Boletaceae, *viz*. Austroboletoideae, Boletoideae, Chalciporoideae, Leccinoideae, Xerocomoideae, Zangioideae, and the *Pulveroboletus* group. This result was widely recognized by other researchers. Lately, Wu et al. unveiled evolutionary innovations from the genomic respects in the ectomycorrhizal Boletales (Wu et al., 2022). The conclusion similar to Kohler et al. (2015) and Miyauchi et al. (2020) showed that Boletales impressively reduced their plant cell wall-degrading enzymes (PCWDEs). However, a phenomenon that different lineages still retained different set of PCWDEs maybe means moderately ability of cell wall-degrading still existed in Boletales.

Although remarkable results on the taxonomy of boletes were obtained in the past (Li et al., 2011; Zeng et al., 2014; Zhao et al., 2014a,b; Gelardi et al., 2015a,b; Zhu et al., 2015; Wu et al., 2016b; Chen et al., 2019; Vadthanarat et al., 2019; Hosen and Yang, 2021; Ayala-Vásquez et al., 2022; Badou et al., 2022; Biketova et al., 2022), the additions of more new taxa are necessary to reconstruct a high-resolution tree of Boletaceae. More intensive collection are needed to analyze the species diversity of Boletaceae in China. Our study is focused on results from our collection in northern China. In this study, we described the morphological and phylogenetic data of a new genus that is evident to form a distinct lineage in the “*Pulveroboletus* group.” Meanwhile, two new species and one new record species of Henan province are also reported.

## Materials and methods

### Samplings and morphological analyses

Specimens were collected from Guizhou and Henan Province, China. Voucher materials were deposited in the Mycology Herbarium of the Jilin Agriculture University (HMJAU). The color of fresh basidiocarps is described following Kornerup and Wanscher’s (1978) method. Tissues of specimens were mounted in 5% KOH and then in 1% Congo Red or Melzer’s solution, and steps of amyloid reactions were followed according to Imler’s procedure (Imler, 1950; Biketova et al., 2022). The observations of microscopic characteristics were performed by Carl Zeiss Lab. A1 optical microscope. The ultrastructure of basidiospores was observed by scanning electron microscope (SEM). Basidiospore dimensions were recorded as length by width, in order of the minimum, the average, and the maximum; the notation (n/m/p) indicated that measurements were made on “p” randomly selected basidiospores from “m” basidiomes of “n” collections. Q is the ratio of length divided by width: Qm = average quotient (length/width ratio) ± standard deviation.

### DNA extraction, PCR amplification, and sequencing

The NuClean Plant Genomic DNA kits (CWBIO) are used to extract genomic DNA. The primers LROR/LR5 were used for *28S*, RPB1-B-F/RPB1-B-R for *rpb1* (Wu et al., 2014), RPB2-B-F1/RPB2-B-R and PRB2-6F/PRB2-7.1R for *rpb2* (Matheny and Ammirati, 2003; Matheny, 2005; Wu et al., 2014), and 983F/1567R for *tef1-*α (Rehner and Buckley, 2005). The polymerase chain reaction (PCR) procedures were executed, referring to Feng et al. (2012) and Wu et al. (2014).

### Phylogenetic analyses

The new sequences were uploaded to NCBI,^1^ and other sequences were downloaded from NCBI (Supplementary Table 1). The raw matrixes (ITS, 28S, *rpb1*, *rpb2*, and *tef1-*α) were spliced in SeqMan (Swindell and Plasterer, 1997) and aligned with MAFFT (Katoh and Standley, 2013) using ‘E-INS-i (accurate)’ strategy and normal alignment mode, respectively. The ITS matrix of *Phylloporus* was aligned by MEGA 7 with the “Muscle” strategy (Kumar et al., 2016). TrimAL v1.2 was used to trim matrixes with the “gappyout” option (Capella-Gutiérrez et al., 2009). Multi-locus datasets were concatenated by PhyloSuite v1.2.1, and phylogenetic trees were constructed by maximum likelihood (ML) and Bayesian inference (BI) analyses (Zhang et al., 2020). In the multi-locus dataset (28S + rpb1 + rpb2 + tef1) of *Hemilanmaoa*, 646 bp for 28S, 761 bp for *rpb*1, 705 bp for *rpb*2, and 621bp for *tef*1. In the four-locus dataset of Porphyrellus E.-J. Gilbert and Lanmaoa G. Wu & Zhu L. Yang, 835 bp for 28S, 611 bp for *rpb*1, 642 bp for *rpb*2, and 620 bp for *tef*1. In the three-locus dataset (ITS + 28S + *tef*1) of Phylloporus Quél. 919 bp for 28S, 586 bp for *tef*1, and 996 bp for ITS. Best models of matrixes were searched using PartitionFinder 2 integrated into PhyloSuite v1.2.1 (Lanfear et al., 2017; Zhang et al., 2020). Models employed for each locus of *Hemilanmaoa* were GTR + I + G for 28S and *rpb*1, SYM+ I + G for *rpb*2 and *tef*1, for the locus of *Porphyrellus* and *Lanmaoa*, GTR + I + G for 28S, SYM + G for *rpb*1, SYM + I + G for *tef*1 and *rpb*2, and the locus of *Phylloporus*, GTR + I + G for 28S and ITS, and K80 + I + G for *tef*1. ML analyses were executed by IQ-tree (Nguyen et al., 2015) using ultrafast bootstrap with 5,000 replicates. BI analyses were executed using MrBayes 3.2.6 (Ronquist et al., 2012), running in 2,000,000 generations, and sampled every 2,000 generations. The initial 25% of the sampled data were discarded as burn-in. Other parameters were kept at default settings.

## Results

### Molecular phylogeny

**FIGURE 1:**
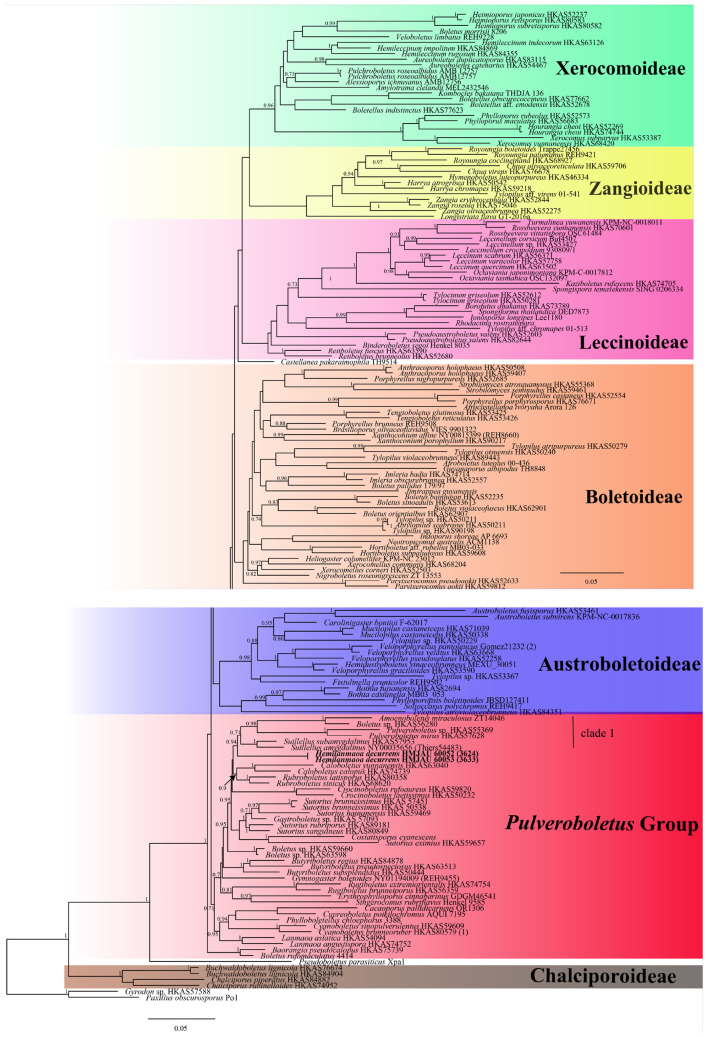
Phylogenetic relationships of *Hemilanmaoa* in Boletaceae inferred from multi-locus (28S, tef1-α, rpb1, and rpb2) using Bayesian inference. PP ≥ 0.7 are indicated in the phylogram. Newly formed sequences in this study are indicated in bold.

**FIGURE 2:**
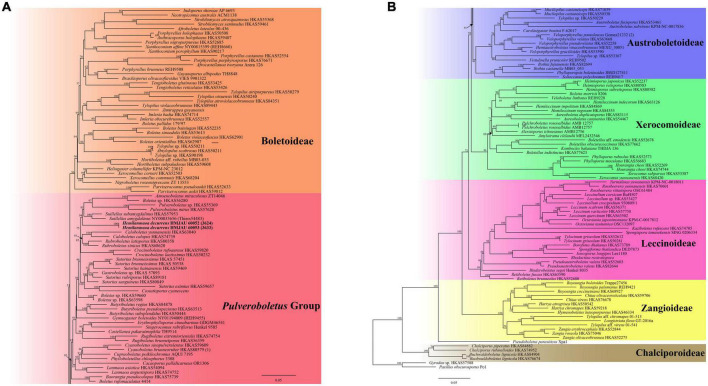
Phylogenetic relationships of *Hemilanmaoa* in Boletaceae inferred from multi-locus (28S, tef1-α, rpb1, and rpb2) using the maximum likelihood method. BP ≥ 50 is indicated in the phylogram. Newly formed sequences in this study are indicated in bold. **(A)** Phylogram upper part. **(B)** Phylogram lower part.

In the phylogenetic relationship of Boletaceae, the Bayesian tree (Figure 1) and ML tree (Figure 2) were listed, respectively, due to some differences between the major clades of the Bayesian tree and ML tree. The multi-locus datasets consisted of 203 taxa and 2,733 nucleotide sites (Figures 1, 2). *Gyrodon* sp. and *Paxillus obscurosporus* C. Hahn were chosen as outgroups. The molecular phylogenetic analyses showed that *Hemilanmaoa retistipitatus* is not only embedded in the “*Pulveroboletus* Group” clade but also sister with *Suillellus* Murrill. It formed an independent lineage with a strong support value (BP = 0.94, PP = 94).

The four-locus datasets of *Porphyrellus* and *Lanmaoa* consisted of 89 taxa and 2,709 nucleotide sites. *Butyriboletus appendiculatus* (Schaeff.) D. Arora & J. L. Frank and *Bu. autumniregius* D. Arora & J. L. Frank were chosen as outgroups. In the phylogram (Figure 9), our sequences W3013, W3029, and W3022 were clustered together with *Lanmaoa angustispora* G. Wu & Zhu L. Yang (BP = 1, PP = 100) and formed an independent lineage. In the *Porphyrellus*, our sequences, namely w3088, w3085, w3054, w3046, w3062, w3019, w3039, and w3091, were clustered together with one previously described *Po. cyaneotinctus* (A.H. Sm. & Thiers) Singer (BP = 1, PP = 100).

**FIGURE 3:**
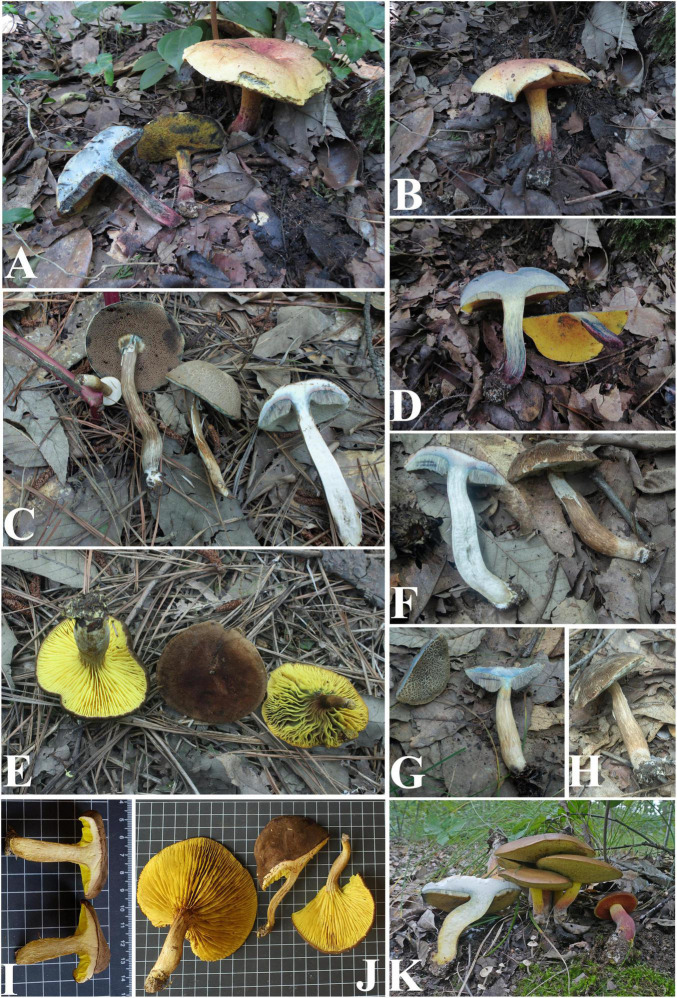
Basidiomata of boletes **(A,B,D)** *Hemilanmaoa retistipitatus*, **(C,F–H)** *Porphyrellus pseudocyaneotinctus*, **(E,I,J)** *Phylloporus biyangensis*, and **(K)** *Lanmaoa angustispora*.

**FIGURE 4:**
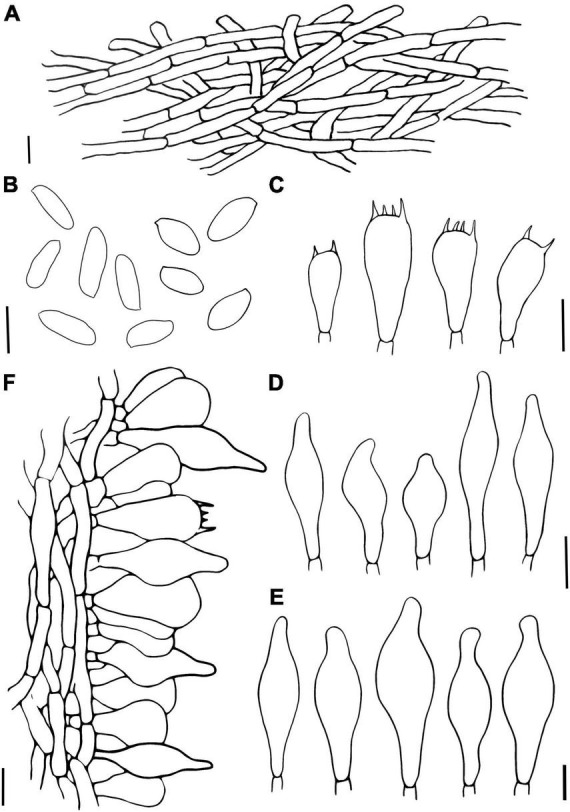
*Hemilanmaoa retistipitatus* **(A)** Pileipellis, **(B)** Basidiospores, **(C)** Basidia, **(D)** Cheilocystidia, **(E)** Pleurocystidia, and **(F)** Stipitipellis. Scale bars = 10 μm.

**FIGURE 5:**
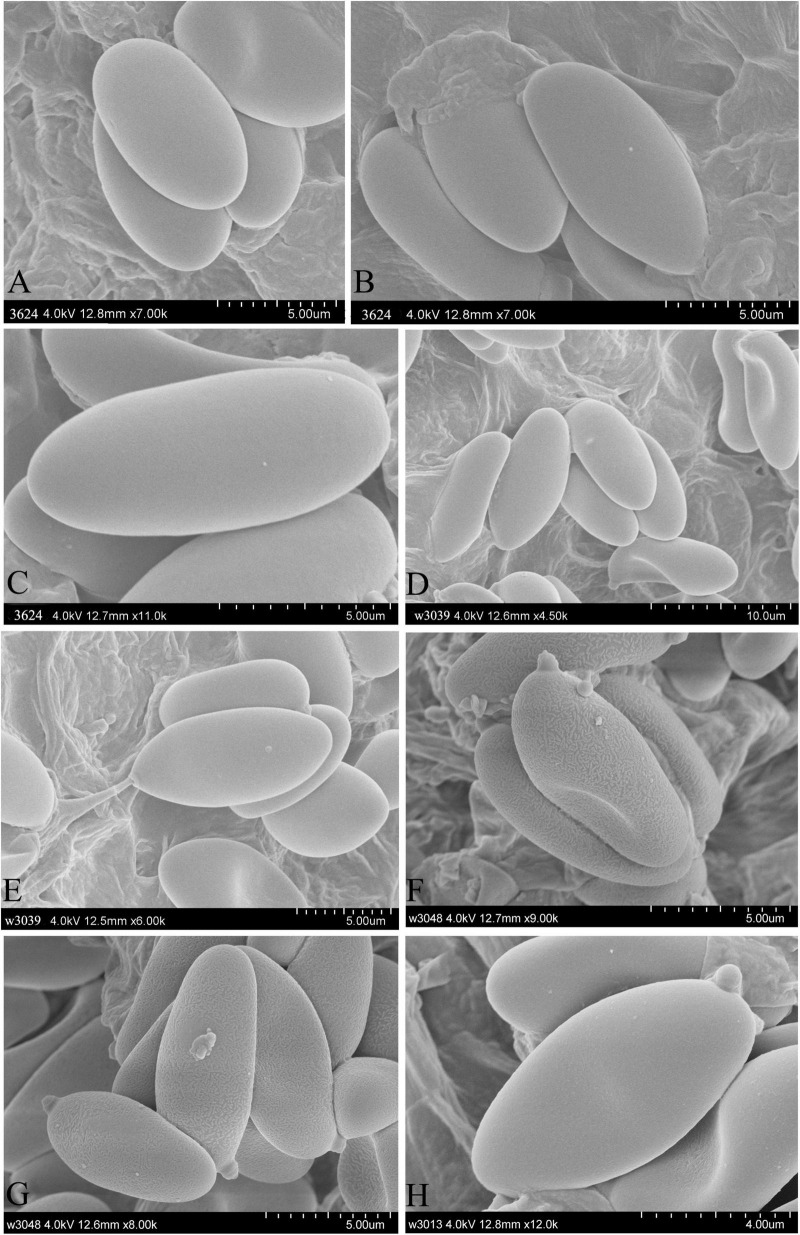
Characteristics of basidiospore in SEM. **(A–C)** *Hemilanmaoa retistipitatus*, **(D,E)** *Porphyrellus pseudocyaneotinctus*, **(F,G)** *Phylloporus biyangensis*, and **(H)** *Lanmaoa angustispora*.

**FIGURE 6:**
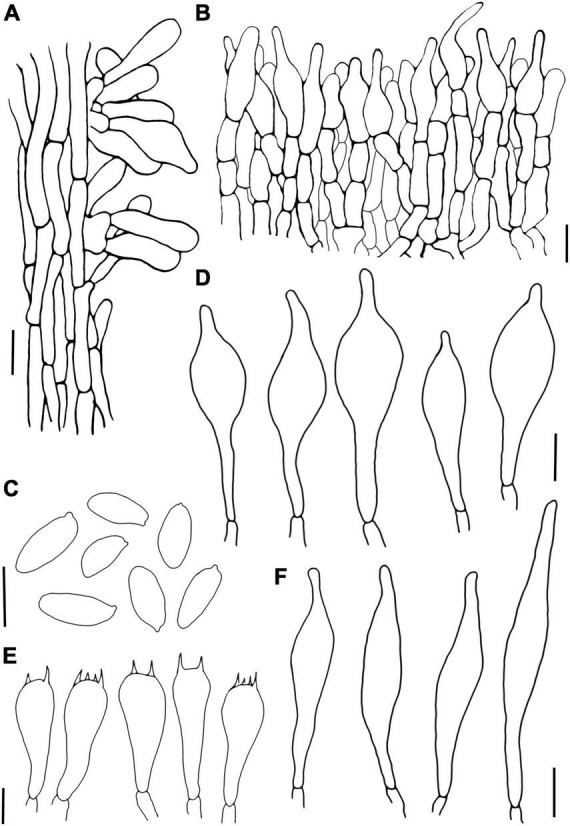
*Porphyrellus pseudocyaneotinctus* **(A)** Stipitipellis, **(B)** Pileipellis, **(C)** Basidiospores, **(D)** Cheilocystidia, **(E)** Basidia, and **(F)** Pleurocystidia. Scale bars = 10 μm.

**FIGURE 7:**
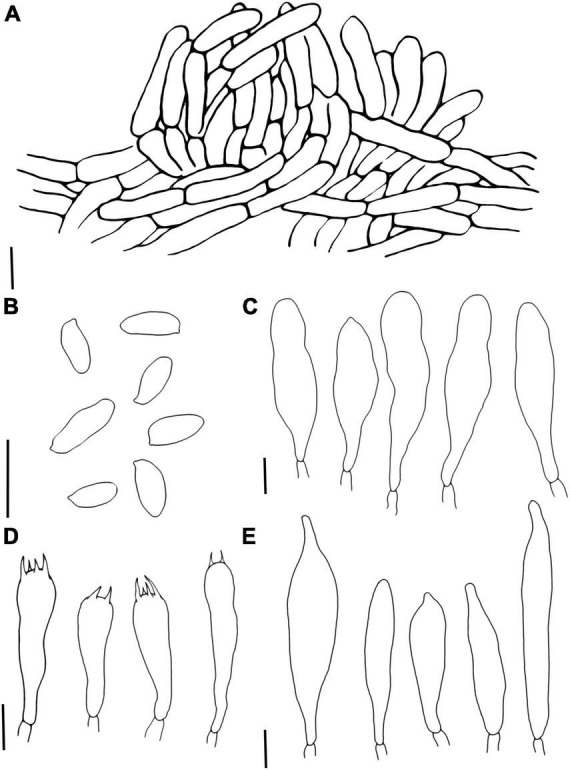
*Phylloporus biyangensis* **(A)** Pileipellis, **(B)** Basidiospores, **(C)** Cheilocystidia, **(D)** Basidia, and **(E)** Pleurocystidia. Scale bars = 10 μm.

**FIGURE 8:**
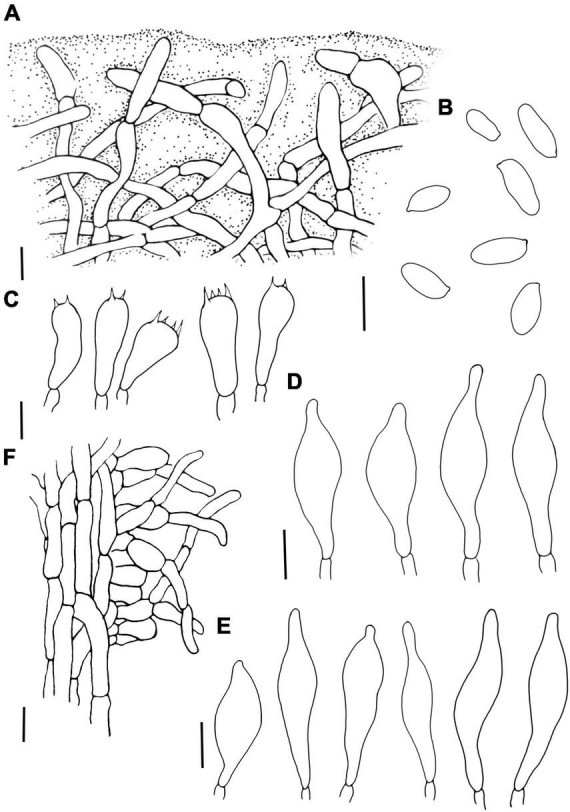
*Lanmaoa angustispora* **(A)** Pileipellis, **(B)** Basidiospores, **(C)** Basidia, **(D)** Cheilocystidia, **(E)** Pleurocystidia, and **(F)** Stipitipellis. Scale bars = 10 μm.

**FIGURE 9:**
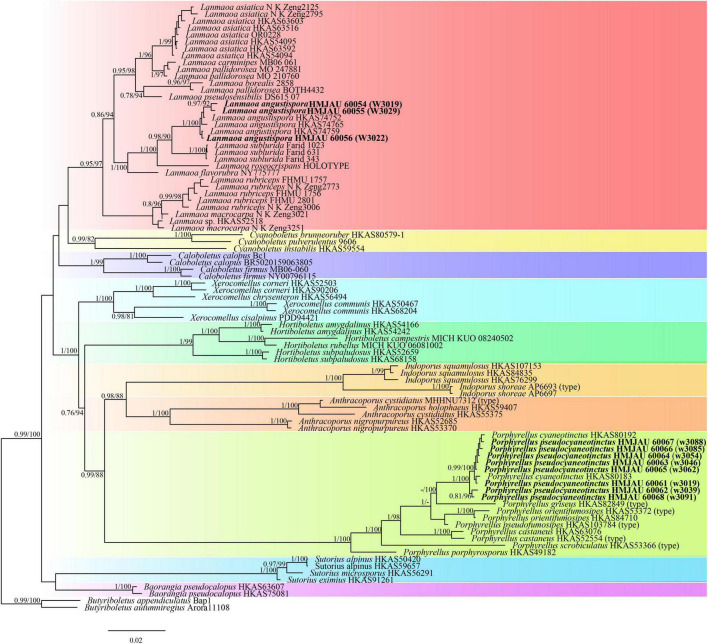
Phylogenetic relationships of *Porphyrellus* and *Lanmaoa* in their genera inferred from multi-locus (28S, tef1-α, rpb1, and rpb2) using the maximum likelihood method and Bayesian inference (only the BI tree was shown). BP ≥ 0.8 and PP ≥ 0.7 are indicated in the phylogram. Newly formed sequences in this study are indicated in bold.

**FIGURE 10:**
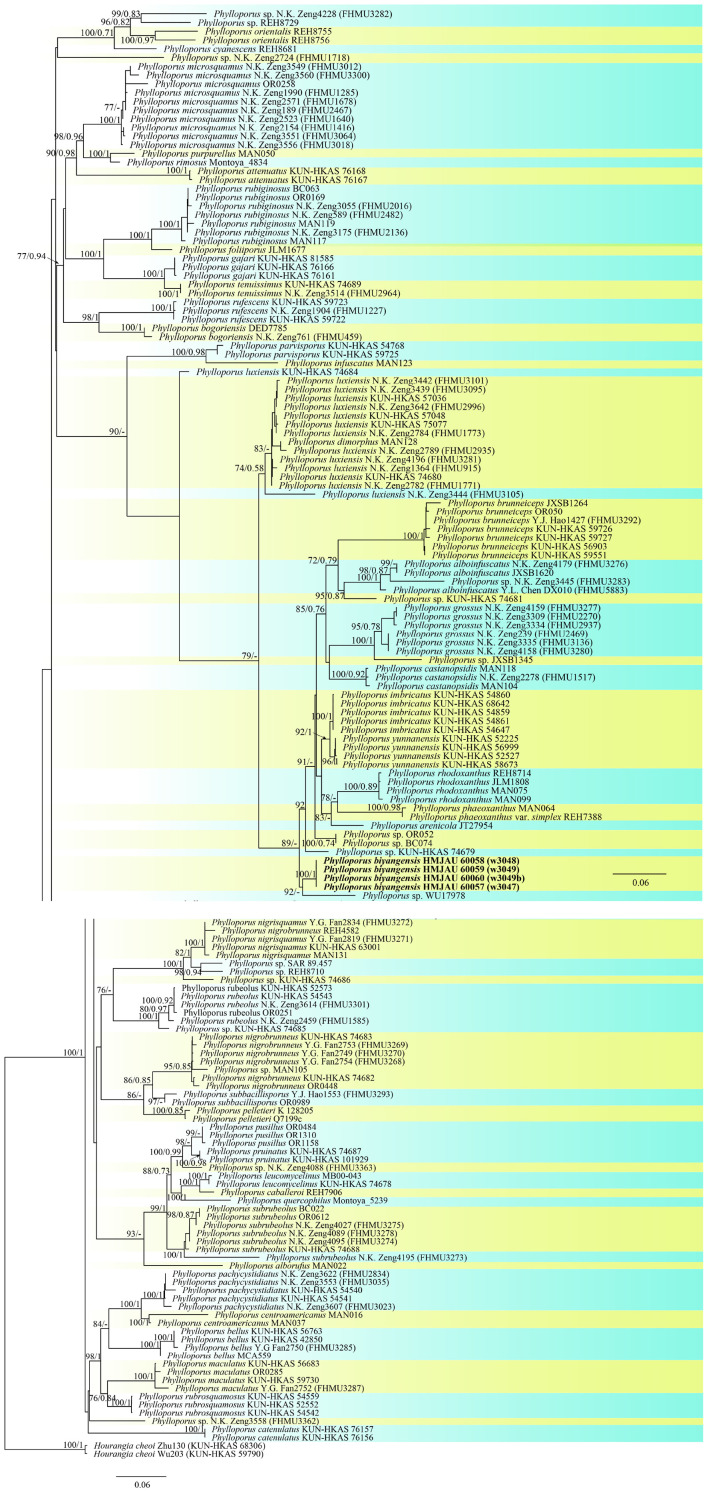
Phylogenetic relationships of *Phylloporus* in the genus inferred from multi-locus (ITS, 28S, and tef1-α) using maximum likelihood method and Bayesian inference (only the ML tree was shown). BP ≥ 0.7 and PP ≥ 0.7 are indicated in the phylogram. Newly formed sequences in this study are indicated in bold.

The three-locus dataset (28S + ITS + tef1) of *Phylloporus* consisted of 170 taxa and 2,501 nucleotide sites. *Hourangia cheoi* (W.F. Chiu) (Xue T. Zhu & Zhu L. Yang) was selected as outgroups. In the phylogram (Figure 10), our sequences w3047, w3048, w3049a, and w3049b formed an independent clade with a high support value (BP = 100, PP = 1).

### Taxonomy

***Hemilanmaoa*** **Yang Wang, Bo Zhang & Y. Li gen. nov.**
Mycobank No.: MB845571
Etymology. “Hemi” refers to its morphological similarity to *Lanmaoa.*

Diagnosis. This genus is similar to *Lanmaoa* but differs from the latter by pores red at the mature, stipe covered with distinctly reticulations and hyphae dextrinoid. Basidioma bluing when bruising, pileus subtomentose, hymenophore decurrent with surface red, stipe covered with reticulations and red dots, and hyphae of context dextrinoid.

Basidioma stipitate-pileate with tubular hymenophore. Pileus hemispherical and depression at the center, subtomentose, dry, margin shortly appendiculate, grayish red to pastel red in the center, pale yellow toward margin; context whitish to pale yellow, discoloring to blue when injured. Hymenophore decurrent, surface orange-red, turning to blue when bruised; pores compound, angular to round, tubes light yellow, changing to blue when cut. Stipe central, yellow at the upper partition, brownish red downwards base, surface reticulate, especially on the upper partition, and erratically covered with brownish red dotted elements, staining blue when touched. Context of stipe brownish red at the base, changing to blue when injured. Basidiospores smooth, ellipsoid, yellowish brown, Pileipellis an interwoven trichodermium. Stipitipellis fertile, caulobasidia scattered. Hyphae of context dextrinoid. Clamp connections absent. Odor mild.

Type species: *Hemilanmaoa retistipitatus* Yang Wang, Bo Zhang & Y. Li
*Hemilanmaoa retistipitatus* Yang Wang, Bo Zhang & Y. Li, sp. nov.
Mycobank No.: MB845573
Figures 3A,B,D, 4, 5A–C
Etymology. “*retistipitatus*” refers to its’ stipe covered with reticulations.
Holotypus. CHINA. Guizhou Province, Tongren City, Yangxi County, 6 July 2019, 108° 30′ 19.35″ E, 27° 38′ 8.16″ N, 3624 (HMJAU 60052!).

Diagnosis. This species is similar to *Lanmaoa macrocarpa* but differs from the latter by decurrent hymenophore, red pores, stipe covered with reticulations, and context dextrinoid. Basidiocarps bluish when bruising, pileus tomentose, hymenophore decurrent with surface red, stipe covered with reticulations and red dots, and hyphae of context dextrinoid.

Basidioma medium-sized. Pileus around 9 cm in diam., hemispherical with an indistinct or distinct depression at the center when mature, sterile margin narrow; grayish red to pastel red (7B5–10A4) in the center, pale yellow (2A3) toward margin; surface dry, subtomentose, discoloring into blue when touched. Context firm, whitish to pale yellow, turning to blue when cut. Hymenophore decurrent, surface orange red (8A7), changing to blue when injured; pores nearly round to angular; tubes 2–13 mm, light yellow (3A5), changing to blue when injured. Stipe 7.9–10.3 × 1.4–1.6 cm, solid, central, subcylindrical to slightly obclavate, pale yellow (2A3) at the upper partition, brownish red (10C6) downwards, surface coarse, covered with yellow reticulations especially on the upper partition and brownish red dotted-elements, staining blue when touched; context concolorous with that of pileus on the upper partition, gradually brownish red downwards, turning to blue when cut; basal mycelium white. Odor mild.

Basidia 16.5–34 × 10–14 μm, subcylindrical to clavate, hyaline to pale brown in 5% KOH, 2- and 4-spored. Basidiospores (2/4/117) 10.0–11.6–13.1 × 4.2–4.9–5.8 (6.0) μm, Q = 2–2.7 (2.9), Qm = 2.37 ± 0.17, ellipsoid, yellowish brown in 5% KOH, smooth. Hymenophoral trama boletoid, composed of hyaline to brownish yellow hyphae, 2–12 μm wide. Cheilocystidia 22–40 × 7–14.8 μm, narrowly lageniform to lageniform, thin-walled, hyaline to pale brownish yellow in 5% KOH. Pleurocystidia 33–50 × 7–14 μm, similar to cheilocystidia in shape. Pileipellis is an interwoven trichodermium, composed of hyaline to yellowish brown and filamentous hyphae, 2.5–6 μm wide. Stipitipellis fertile, a hymeniform, with inflated terminal cells, ovoid to obovoid, 22–25 × 12–15 μm, hyaline to brownish yellow, caulocystidia 20.2–53 × 8–14.2 μm, lageniform to broadly lageniform, hyaline to pale brownish yellow, caulobasidia scattered. Context dextrinoid in Melzer’s, especially hyphae of stipe base. Clamp connections absent.

Habitat. Solitary or scattered in a mixed broad-leaf forest, dominated by *Cyclobalanopsis* sp. and Lauraceae.

Distribution. Currently, only known in Guizhou Province, China.

Additional specimens measured. CHINA. Guizhou Province, Tongren City, Yangxi County, 6 July 2019, 108° 30′ 19.35″ E, 27° 38′ 8.16″ N, 3633 (HMJAU 60053).

Notes. *Lanmaoa macrocarpa* shares some morphological features with *He. retistipitatus*, *viz*. similar color of pileus surface and stipe, bluing when injured. However, it can be distinguished by its hymenophore depressed around the apex of the stipe, tubes concolorous with pore surfaces, basal mycelium yellowish, smaller basidiospores (10–12 × 4.5–5 μm), pileipellis a trichoderm and without reaction in Melzer’s.

*Hemilanmaoa retistipitatus* resembles *Cyanoboletus cyaneitinctus* (Murrill) A. Farid, A. R. Franck & J. A. Bolin in its reddish color toward stipe base, decurrent hymenophore, and bluing strongly when handled. However, the latter taxon has a duller pileus, yellow hymenophore, basidiospores larger (11.5–15 × 4–6 μm), and stipitipellis sterile.

*Porphyrellus pseudocyaneotinctus* Yang Wang, Bo Zhang & Y. Li, sp. nov.
MycoBank No.: MB845570
Figures 3C,F–H, 5D,E, 6

Etymology. The epithet “*pseudocyaneotinctus*” refers to its similarity to *Po*. *cyaneotinctus.*

Holotypus. CHINA. Henan Province, Zhumadian City, Biyang County, 9 July 2021, W3039 (HMJAU 60062!).

Diagnosis. This species is similar to *Po*. *cyaneotinctus*, but differs from the latter by different structures of pileipellis, without reaction in Melzer’s and thin-walled caulocystidia with no thickening in the apex. Basidioma brown, often with distinctly cracked pileus, spores broader, and caulocystidia thin-walled.

Basidioma small to medium-sized. Pileus 4.6–9.9 cm wide, subhemispherical to convex or subconvex, caramel (6C6) to light brown (6D6) or brown (6E4), slightly darker in the center; surface dry, tomentose, with finely or distinctly cracked, sometimes with sterile margin at mature; context 0.5–1.2 cm thick, white (3A1), erratically bluish then reddish brown when injured. Hymenophore adnexed or depressed around the apex of stipe with finely decurrent tooth; surface white (3A1) when young, pale gray (1B1) or light brown (6D8) at the mature, becoming blue when bruised; pores angular, 0.75–3/mm; tubes 0.45–1.70 cm long, concolorous with or a little duller than the hymenophoral surface, changing to blue when injured. Stipe 4.8–12.3 × 0.9–1.9 cm, subcylindrical, sometimes slightly expanded or attenuate to base, concolorous with the pileus, streaked, fibrillose, context white (3A1), sometimes nougat (5D3) at the base, usually turning to red-brown when bruised, changing to blue at the apex when cut; basal mycelium white.

Basidiospores (12/12/150) 9.8–11.8–13.8 (14.5) × 4.5–5.3–6.2 μm, Q = 1.83–2.79, Qm = 2.24 ± 0.19, ellipsoid to elongate ellipsoid, inequilateral with a suprahilar depression in side view, light yellow to reddish brown in 5% KOH, smooth. Basidia 24–42 × 9.9–16.2 μm, clavate, 2-, 4-spored, hyaline in 5% KOH, hyaline to yellow in Melzer’s. Hymenophoral trama boletoid consists of 6–17.5 μm wide hyphae. Pleurocystidia 36.8–85 × 8.5–13 μm, lageniform, hyaline in 5% KOH and Melzer’s. Cheilocystidia 38.2–60.5 × 10.8–17.9 μm, similar to pleurocystidia in shape, hyaline in 5% KOH and Melzer’s. Pileipellis a palisadodermium, composed of broadly concatenated cells, hyaline, sometimes brownish in 5% KOH, terminated cells filamentous or pyriform to subfusiform, 20.5–92.5 × 5–23.8 μm, the lower 2–3 cells broad, 26.3–60 × 12.5–25 μm. Stipitipellis sterile, hymeniform with thin-walled and inflated terminal cells, 16.8–49 × 5–18 μm, without a hyaline (in 5% KOH) refractive thickening in the apex of the cells. Clamp connection absent.

Habitat. Solitary on mixed forests dominated by *Quercus* spp. and *Pinus* spp.
Known distribution. Currently, only known from Henan Province, China.

Additional collection examined. CHINA. Henan Province, Zhumadian City, Biyang County, Tongshan Lake, 8 July 2021, 113° 29′ 44.48″ E, 32° 46′ 8.32″ N, W3019 (HMJAU 60061), 9 July 2021, 113° 29′ 47.40″ E, 32°46′9.76″ N, W3046 (HMJAU 60063); Baiyun Mountain, 10 July 2021, 113° 34′ 9.57″ E, 32° 53′ 19.70″ N, W3054 (HMJAU 60064), W3062 (HMJAU 60065); Baiyun Mountain, 11 July 2021, 113° 34′ 0.86″ E, 32° 53′ 19.51″ N, W3083, W3084, W3085 (HMJAU 60066), W3088 (HMJAU 60067), W3091 (HMJAU 60068), W3092.

Notes. Phylogenetically, *Porphyrellus pseudocyaneotinctus* is a sister of *Po. griseus* Yan C. Li & Zhu L. Yang and *Po*. *pseudofumosipes* Yan C. Li & Zhu L. Yang. However, *Po. griseus* differs from *Po. pseudocyaneotinctus* in the context of pileus turning to blue when injured, basidiospores smaller (9.5–11.5 × 4.5–5 μm), pleurocystidia shorter (34–58 × 8–12 μm), terminal cells of pileipellis broadly clavate to cystidioid or pyriform. *Porphyrellus pseudofumosipes* is different from *Po*. *pseudocyaneotinctus* in context of pileus bluish when injured, basidiospores smaller (9–11 × 4.5–5.5 μm), and terminal cells of pileipellis pyriform to subfusiform. Morphologically, *Porphyrellus pseudocyaneotinctus* is similar to *Po*. *castaneus* Y.C. Li & Zhu L. Yang, *Po*. *cyaneotinctus* (A.H. Sm. & Thiers) Singer, *Po*. *formosanus* K.W. Yeh & Z.C. Chen, *Po*. *orientifumosipes* Y.C. Li & Zhu L. Yang, and *Po*. *pseudofumosipes* Yan C. Li & Zhu L. Yang. However, *Po*. *castaneus* is characterized by its duller pileus, bluish when injured, basidiospores smaller (9–11 × 4–5 μm), terminal cells of pileipellis pyriform or subfusiform. *Porphyrellus cyaneotinctus* differs from *Po*. *pseudocyaneotinctus* in the upper 1-3 cells of pileipellis more or less cylindric and 5–9 μm wide, the lower 3-5 cells of pileipellis somewhat to distinctly inflated (9–25 μm wide), pleurocystidia with dark yellow-brown content in Melzer’s or more rarely with strongly amyloid granules, caulocystidia with a hyaline (in KOH) refractive thickening in the apex of the cell that is often forming a lens or cap and grows on poor sandy soil on hillsides with scattered *Quercus* spp. trees during the late summer and autumn. *Porphyrellus formosanus* is characterized by its context of pileus turning red when cut, basidiospores larger (14–25 × 5–6.5 um), and pileipellis consisting of filamentous hyphae. *Porphyrellus orientifumosipes* is different from *Po. pseudocyaneotinctus* in its pileus context bluish when injured, basidiospores 9–11 × 4.5–5.5 μm, terminal cells of pileipellis pyriform to subfusiform. *Porphyrellus pseudofumosipes* differs from *Po. pseudocyaneotinctus* in its pileus context bluish when injured, basidiospores smaller (9–11 × 4.5–5.5 μm), terminal cells of pileipellis 26–57 × 9–16 μm.

*Phylloporus biyangensis* Yang Wang, Bo Zhang & Y. Li, sp. nov.
MycoBank No.: MB845572
Figures 3E,I,J, 5F,G, 7
Etymology. “*biyangensis*” refers to its type locality Biyang County.
Holotypus. CHINA. Henan Province, Zhumadian City, Biyang County, 9 July 2021, W3049 (HMJAU 60059!).

Diagnosis. This species is close to *Ph*. *luxiensis* but differs from the latter by yellowish context, smaller spores, larger cheilocystidia, hyaline to pale brown hyphae of pileipellis. Basidioma dull, context yellowish, hymenophore usually discolored when bruising, basidiospores smaller and hyphae of pileipellis sometimes with granular encrustations.

Basidioma medium-sized. Pileus 7.2–11.9 cm in diam., applanate to slightly depressed at center, margin involute when young; surface dry, tomentose, sometimes cracked into squamulose, reddish brown (8E4–8E7). Context approximately 0.4 cm thick at the position halfway to pileus center, yellow (2A6), color unchanging when injured; Hymenophore lamellate, decurrent, 0.6–0.8 mm broad, subdistant, anastomosing, greenish-yellow (1A8), turning blue or unchanging in color when injured; lamellulae common, concolorous with lamellae. Stipe 3.8–4.7 × 0.6–1.3 cm, central, subcylindrical, tapered downwards base, covered with finely reddish brown squamules; ridged along with decurrent lines of lamellae on the upper partition, context light yellow (4A5), color unchanged when injured. Basal mycelium yellow.

Basidiospores (2/3/124) (6.8) 7–9.47–10.5 (11) × (3.5) 3.8–4.15–4.5 (5.5) μm, Q = (1.56) 1.78–2.63 (2.75), Qm = 2.29 ± 0.23, brownish yellow in 5% KOH, ellipsoid, with a suprahilar depression in side view, smooth under a light microscope but with bacillate ornamentation in scanning electron microscope. Basidia 27.2–44 × 6–11.5 μm, 2–, 4–spored, subcylindrical, hyaline in 5% KOH. Hymenophoral trama phylloporoid, composed of hyphae 4.8–20 μm in diameter. Pleurocystidia 41.5–130 × 10–30 μm, lageniform, subfusiform or subclavate, hyaline to pale brown in 5% KOH. Cheilocystidia 58.5–101 × 12–20.5 μm, similar to pleurocystidia in shape, hyaline to pale brown in 5% KOH. Pileipellis an interwoven trichodermium, consists of filamentous hyphae, 6.3–9.5 μm wide, hyaline to pale brown in 5% KOH, sometimes with granular encrustations. Clamp connection absent.

Habitat. Solitary on mixed forests dominated by *Quercus* spp. and *Pinus* spp.
Known distribution. Currently, only known in Henan Province, China.

Additional collections examined. CHINA. Henan Province, Zhumadian City, Biyang County, Tongshan Lake, 9 July 2021, 113° 29′ 47.95″ E, 32° 46′ 8.89″ N, W3047 (HMJAU 60057), W3048 (HMJAU 60058), W3049b (HMJAU 60060).

Notes. *Phylloporus luxiensis* is similar to *Ph*. *biyangensis* and *Ph*. *bellus* in morphological characteristics. However, *Ph*. *luxiensis* is characterized by its white context, and larger basidiospores (10–12 × 4.5–5 μm) (Zeng et al., 2011). *Phylloporus bellus* differs from *Ph*. *biyangensis* in basal mycelium white, spores longer, and cystidia smaller with encrusting pigment sometimes present.

*Lanmaoa angustispora* G. Wu & Zhu L. Yang
Figures 3K, 5H, 8

Collection. CHINA. Henan Province, Zhumadian City, Biyang County, 8 July 2021, W3013 (HMJAU 60054).

Basidioma medium to large. Pileus 4.6–11.3 cm wide, plano-convex to convex, reddish brown (8E8) when young, light brown (6D7) to brownish yellow (5C8), surface nearly smooth, incurved at the margin; context 1.0–2.1 cm thick, soft, pastel green (29A4) to white (1A1), staining blue immediately when injured. Hymenophore adnexed with a decurrent tooth around stipe or sinuate, surface orange-red (8A6) to Persian orange (6A7) when young, greenish-yellow (1B6) to yellow (2A6) at mature, staining blue when bruised; pores angular to nearly round, 1–2/mm; tubes short, 0.6–1.0 cm long, greenish-yellow (1B6) to yellow (2A6), staining blue when injured. Stipe 6.2–13 × 1–1.8 cm, subcylindrical, greenish-yellow (1A8) at the apex, pastel red (9A5) toward the base, covered with red dots; context yellowish green (30A8), changing to blue when injured. Basal mycelium white.

Basidiospores (1/2/60) 9.00–10.10–11.50 (15.10) × 3.50–3.96–5.00 μm, Q = 2.25–3.02, Qm = 2.55 ± 0.17, subcylindrical to elongate ellipsoid, inequilateral in side view with slightly suprahilar depression, yellow-brown in 5% KOH, smooth. Basidia 20.5–34.0 × 7.2–11.0 μm, clavate, hyaline, or pale brown in 5% KOH. Hymenophoral trama boletoid type consists of 7.5–14.5 μm wide hyphae. Pleurocystidia 29.2–57.8 × 8.2–16.6 μm, lageniform, thin-walled, brown in 5% KOH. Cheilocystidia 25.0–45.9 × 6.8–14.5 μm, similar to pleurocystidia in shape, thin-walled, usually containing yellow to brownish-yellow pigments. Pileipellis interwoven ixotrichodermium consists of hyaline filamentous hyphae 5–7.5 μm in width, with terminal cells subcylindrical or sometimes with subacute apex. Stipitipellis composed of two layers, outer layer trichodermium, consisting of hyaline interwoven filamentous hyphae; inner layer hymeniform, with 16–25 × 8.8–13 μm thin-walled and inflated terminal cells. Clamp connection not observed.

Habitat. Scattered on the sandy soil in *Castanea* spp. forest.
Known distribution. Known to be distributed in southwestern China and central China.

Additional collections were examined. CHINA. Henan Province, Zhumadian City, Biyang County, 8 July 2021, 113° 29′ 45.25″ E, 32° 46′ 8.62″ N, Q024), W3022 (HMJAU 60056), 9 July 2021, 113° 29′ 47.40″ E, 32° 46′ 9.76″ N, W3029 (HMJAU 60055).

Notes. The *hymenophoral* characteristics of *Lan. angustispora* are different from the original description by Wu et al. (2016b) in hymenophoral surface is reddish when young, and yellow at mature. Morphologically, *Lan*. *angustispora* resembles *Lan*. *flavorubra* and cluster together in the phylogenetic tree; however, stipe of *Lan*. *angustispora* smooth, context soft and spores narrower.

## Discussion

Macrofungi in East Asia are well-known to mycologists, because of their highly diverse and endemic nature (Hongo, 1960; Horak et al., 2011; Feng et al., 2012; Zeng et al., 2013; Wu et al., 2016a; Cui et al., 2019). The discovery of the new genus *Hemilanmaoa* further demonstrated this circumstance. Phylogenetically, *Hemilanmaoa* is embedded in the *Pulveroboletus* Group, forming a distinct lineage. *Hemilanmaoa* is the sister of “clade 1” (Figure 1), including *Amoenoboletus* G. Wu, E. Horak, & Zhu L. Yang, *Pulveroboletus* Murrill, and *Suillellus* However, *Hemilanmaoa* is different from *Amoenoboletus* in its context, and hymenophores change to blue when injured (Chen et al., 2019); *Pulveroboletus* differs in its pulverulent surface, distinctly marginal veil, and adnexed hymenophore (Wu et al., 2016a; Zeng et al., 2017); *Suillellus* can be distinguished by its usually olive pileus surface and amyloid hyphae of stipe base (Vizzini et al., 2014; Wang et al., 2022). Morphologically, *Hemilanmaoa* is similar to *Rubroboletus* Kuan Zhao & Zhu L. Yang, *Neoboletus* Gelardi, Simonini & Vizzini, *Caloboletus* Vizzini, *Cyanoboletus* Gelardi, Vizzini & Simonini and *Lanmaoa* G. Wu & Zhu L. Yang. However, *Rubroboletus* differs from *Hemilanmaoa* in its pileal surface reddish and pink to red reticulations on the surface of the stipe (Zhao et al., 2014b); *Neoboletus* is different from *Hemilanmaoa* due to its smooth stipe with no reticulations (Wu et al., 2016a); *Caloboletus* can be distinguished by paler pileus and smooth or reticulate stipe without dots (Wu et al., 2016a); *Cyanoboletus* is characterized by its stipe pruinose to the furfuraceous surface, ixosubcutis to subcutis pileipellis (Wu et al., 2016a); *Lanmaoa* differs from *Hemilanmaoa* in its adnexed or sinuate hymenophore and much thinner hymenophore (Wu et al., 2016b; Chai et al., 2019).

In our study, the phylogenetic analyses of Boletaceae showed different topologies in the BI and ML trees. None is similar to Wu et al. (2016a). The order of seven major clades in the BI tree was Chalciporoideae, *Pseudoboletus*, *Baorangia*, *Pulveroboletus* group, Austroboletoideae, Boletoideae, Leccinoideae, Zangioideae, and Xerocomoideae. However, the order in the ML tree was Chalciporoideae, *Pseudoboletus*, Zangioideae, Leccinoideae, Xerocomoideae, Austroboletoideae, *Pulveroboletus* group, and Boletoideae, and *Baorangia* clade was embed in *Pulveroboletus* group. In view of our results and combined with phylogenetic analyses of Zhao et al. (2014b), Badou et al. (2022), and Vadthanarat et al. (2022), the positions of the several major clades are still unstable. To confirm the order of the clades formed, more different species and more genes should be introduced to construct the phylogenetic tree. The phylogenetic trees (Figures 1, 2) showed that some sequestrate boletes were not monophyletic groups, such as *Turmalinea* Orihara & N. Maek., and *Heliogaster* Orihara & K. Iwase. *Turmalinea* and *Heliogaster* were paraphyletic with *Rossbeevera* T. Lebel & Orihara and *Xerocomellus* Šutara, respectively. However, in the study of Orihara et al. (2016), *Turmalinea* was the sister of *Rossbeevera* and formed an independent lineage. Given the extremely affair phylogenetic relationships between *Turmalinea* and *Rossbeevera*, we are suspicious about the reliability of separating *Turmalinea* from *Rossbeevera.* However, the further conclusion needs more detailed and abundant information on species.

In the phylogenetic analyses of *Porphyrellus* and *Lanmaoa*, our new species clustered with *Po*. *cyaneotinctus* (A.H. Sm. & Thiers) Singer. It was first published by Smith and Thiers based on specimens from Michigan and was now re-described by Li et al. according to Chinese collections in 2022 (Smith and Thiers, 1968; Li and Yang, 2021). Similar to Li et al., we also collected specimens from Henan Province, China with similar habitats. Combined with morphological characteristics and phylogenetic results, we recognized that this clade represented one species. However, compared to the original descriptions of *Po*. *cyaneotinctus*, there are four main characteristics, *viz*. (1) a different pileipellis structure, (2) color of pleurocystidia in Melzer’s, (3) features of caulocystidia, and (4) different habitat. Taken all together, they are sufficient to warrant the elevation of the *Po*. *pseudocyaneotinctus.*

In the phylogenetic analyses of *Phylloporus*, the “*Phylloporus luxiensis* KUN-HKAS 74684” clade was separated from the major clade of “*Phylloporus luxiensis*,” which was different from the result of Wu et al. (2021) but similar to that of Zeng et al. (2013).

## Data availability statement

The data presented in this study are deposited in the Zenodo repository, accession number 10.5281/zenodo.7538325.

## Author contributions

YL and BZ: conceptualization. YW: methodology, writing—original draft preparation, and formal analysis. L-YW, Z-XQ, DD, Z-HZ, Y-JL, J-JH, and PZ: investigation. All authors contributed to the article and approved the submitted version.

## Footnotes

1
https://www.ncbi.nlm.nih.gov/
