# Supplementary material for: Boletaceae in China: Taxonomy and phylogeny reveal a new genus, two new species, and a new record
Source: Front Microbiol. 2023 Feb 2;13:1052948. doi: 10.3389/fmicb.2022.1052948 (PMC9932287; doi:10.3389/fmicb.2022.1052948)
Supplement: Supplementary file 2 [file Table_1.docx]

**Table 1.** Information of DNA sequences used to reconstruct phylogenetic trees. Sequences newly generated in this study are indicated in bold.

| **Taxon** | **Voucher ID** | **ITS** | **28S** | ***TEF*1** | ***RPB*1** | ***RPB*2** | **Origin** | **References** |
| --- | --- | --- | --- | --- | --- | --- | --- | --- |
| ***Hemilanmaoa retistipitatus*** | **HMJAU 60052** | **–** | **OP380695** | **OP495816** | **OP495812** | **OP495814** | **China** | **This study** |
| ***Hemilanmaoa retistipitatus*** | **HMJAU 60053** | **–** | **OP380696** | **OP495817** | **OP495813** | **OP495815** | **China** | **This study** |
| *Abtylopilus scabrosus* | HKAS50211 | – | KT990552 | KT990752 | KT990920 | KT990389 | China | Wu et al., 2016a |
| *Afroboletus luteolus* | 00-436 | – | KF030238 | KF030397 | KF030392 | – | Africa | Nuhn et al., 2013 |
| *Afrocastellanoa ivoryana* | Arora 126 | – | KX685721 | KX685715 | – | – | Africa | Orihara and Smith, 2017 |
| *Alessioporus ichnusanus* | AMB12756 | – | KJ729504 | KJ729513 | — | — | Italy | Gelardi et al., 2014a |
| *Amoenoboletus miraculosus* | ZT14046 | – | MW520188 | MW566745 | – | – | Malaysia | Chen et al., 2019 |
| *Amylotrama clelandii* | MEL2432546 | – | MT459235 | MN413630 | – | – | Australia |  |
| *Anthracoporus cystidiatus* | HKAS55375 | – | KT990622 | KT990816 | KT990969 | MT110410 | China | Wu et al., 2016a |
| *An. cystidiatus* | MHHNU7312 type | – | MT154710 | – | MT110377 | MT110411 | China | Li and Yang, 2021 |
| *An. holophaeus* | HKAS50508 | – | KF112465 | KF112244 | KF112553 | – | China | Wu et al., 2014 |
| *An. holophaeus* | HKAS59407 | – | KT990708 | KT990888 | KT991030 | KT990506 | China | Wu et al., 2016a |
| *An. holophaeus* | HKAS59407 | – | KT990708 | KT990888 | KT991030 | KT990506 | China | Wu et al., 2016a |
| *An. nigropurpureus* | HKAS52685 | – | KT990627 | KT990821 | KT990973 | KT990459 | China | Wu et al., 2016a |
| *An. nigropurpureus* | HKAS53370 | – | KT990628 | KT990822 | KT990974 | KT990460 | China | Wu et al., 2016a |
| *Aureoboletus catenarius* | HKAS54467 | – | KT990510 | KT990711 | — | KT990349 | China | Wu et al., 2016a |
| *Au. duplicatoporus* | HKAS83115 | – | KT990512 | KT990713 | KT990892 | KT990351 | China | Wu et al., 2016a |
| *Austroboletus fusisporus* | HKAS53461 | – | KF112486 | KF112214 | KF112572 | KF112767 | China | Wu et al., 2014 |
| *Aus. subvirens* | KPM-NC-0017836 | – | JN378518 | JN378458 | – | – | Japan | Orihara et al., 2012 |
| *Baorangia pseudocalopus* | HKAS75739 | – | KJ184558 | KJ184570 | KJ184564 | KM605179 | China | Wu et al., 2016b |
| *Ba. pseudocalopus* | HKAS63607 | – | KF112355 | KF112167 | – | – | China | Wu et al., 2014 |
| *Ba. pseudocalopus* | HKAS75081 | – | KF112356 | KF112168 | – | – | China | Wu et al., 2014 |
| *Binderoboletus segoi* | Henkel 8035 | – | LC043078 | – | LC043079 | – | Guyana | Henkel et al., 2016 |
| *Boletellus* aff. *emodensis* | HKAS52678 | – | KF112426 | KF112305 | KF112621 | KF112757 | China | Wu et al., 2014 |
| *Bo. indistinctus* | HKAS77623 | – | KT990531 | KT990733 | – | KT990371 | China | Wu et al., 2016a |
| *Bo. obscurecoccineus* | HKAS77662 | – | – | – | – | KT990372 | China | Wu et al., 2016a |
| *Boletus bainiugan* | HKAS52235 | – | KF112457 | KF112203 | KF112587 | KF112705 | China | Wu et al., 2014 |
| *Bol. morrisii* | 8206 | – | KF030326 | KF030433 | – | – | USA | Nuhn et al., 2013 |
| *Bol. orientialbus* | HKAS62907 | – | JN563856 | – | JN563873 | – | China | Feng et al., 2012 |
| *Bol. rufomaculatus* | 4414 | – | KF030248 | KF030406 | KF030369 | – | USA | Nuhn et al., 2013 |
| *Bol. sinoedulis* | HKAS53613 | – | – | – | KM820822 | – | China | Cui et al., 2016 |
| *Boletus* sp. | HKAS56280 | – | KF112418 | KF112265 | KF112541 | KF112708 | China | Wu et al., 2014 |
| *Boletus* sp. | HKAS59660 | – | KF112358 | KF112153 | KF112503 | KF112664 | China | Wu et al., 2014 |
| *Boletus* sp. | HKAS63598 | – | KF112317 | KF112152 | KF112502 | KF112663 | China | Wu et al., 2014 |
| *Bol. violaceofuscus* | HKAS62901 | – | JN563860 | – | JN563877 | – | China | Feng et al., 2012 |
| *Borofutus dhakanus* | HKAS73789 | – | JQ928616 | JQ928576 | JQ928586 | JQ928597 | Bangladesh | Hosen et al., 2013 |
| *Bothia castanella* | MB03_053 | – | DQ867117 | KF030421 | KF030382 | – | USA | Nuhn et al., 2013 |
| *Bot. fujianensis* | HKAS82694 | – | KM269193 | KM272860 | – | – | China | Zeng et al., 2015 |
| *Brasilioporus olivaceoflavidus* | VIES 9901322 | – | OM068912 | – | – | – | Brazil | Magnago et al., 2022 |
| *Buchwaldoboletus lignicola* | HKAS84904 | – | KT990538 | KT990740 | – | KT990377 | Germany | Wu et al., 2016a |
| *Bu. lignicola* | HKAS76674 | – | KF112350 | KF112277 | KF112642 | KF112819 | China | Wu et al., 2014 |
| *Butyriboletus appendiculatus* | Bap1 | – | AF456837 | JQ327025 | – | – | Germany | Wu et al., 2014 |
| *But. autumniregius* | Arora11108 | – | KC184424 | – | – | – | USA | Arora and Frank, 2014 |
| *But. pseudospeciosus* | HKAS63513 | – | KT990541 | KT990743 | KT990909 | KT990380 | China | Wu et al., 2016a |
| *But. regius* | HKAS84878 | – | MT264910 | MT269659 | MT269660 | MT269661 | Germany | Wu Kui et al. 2020 |
| *But. subsplendidus* | HKAS50444 | – | KT990540 | KT990742 | KT990908 | KT990379 | China | Wu et al., 2016a |
| *Cacaoporus pallidicarneus* | OR1306 | – | – | MK372272 | – | MK372285 | Thailand | Vadthanarat et al., 2019 |
| *Caloboletus calopus* | HKAS74739 | – | KF112335 | KF112166 | KF112507 | KF112667 | China | Wu et al., 2014 |
| *Ca. calopus* | Bc1 | – | AF456833 | JQ327019 | – | – | Germany | Halling et al., 2012a |
| *Ca. calopus* | BR5020159063805 | – | KJ184554 | KJ184566 | – | – | Belgium | Zhao et al., 2014a |
| *Ca. firmus* | MB06-060 | – | – | KF030408 | KF030368 | – | USA | Nuhn et al., 2013 |
| *Ca. firmus* | NY00796115 | – | KJ605678 | KJ619464 | – | – | USA | Zhao et al., 2014a |
| *Ca. yunnanensis* | HKAS63040 | – | KJ605676 | KJ619471 | KJ619480 | KT990395 | China | Wu et al., 2016a |
| *Carolinigaster bonitoi* | F-62017 | – | NG_068854 | – | – | – | USA |  |
| *Castellanea pakaraimophila* | TH9514 | – | KC155381 | – | – | – | Guyana | Smith et al., 2013 |
| *Chalciporus piperatus* | HKAS84882 | – | KT990562 | KT990758 | – | KT990397 | Germany | Wu et al., 2016a |
| *Ch. rubinelloides* | HKAS74952 | – | KT990565 | KT990761 | — | KT990400 | China | Wu et al., 2016a |
| *Chiua olivaceoreticulata* | HKAS59706 | – | KT990593 | KT990787 | KT990941 | KT990428 | China | Wu et al., 2016a |
| *Chi. virens* | HKAS76678 | – | KF112438 | KF112272 | KF112582 | KF112793 | China | Wu et al., 2014 |
| *Costatisporus cyanescens* |  | – | – | – | LC053663 | LC053664 | Guyana | Smith et al., 2015 |
| *Crocinoboletus laetissimus* | HKAS50232 | – | KT990567 | KT990762 | KT990925 | – | China | Wu et al., 2016a |
| *Cr. rufoaureus* | HKAS59820 | – | KF112434 | – | KF112532 | KF112709 | China | Wu et al., 2016a |
| *Cupreoboletus poikilochromus* | AQUI 7195 | – | KT157061 | – | – | – | Italy | Gelardi et al., 2015a |
| *Cyanoboletus brunneoruber* | HKAS80579 1 | – | KT990568 | KT990763 | KT990926 | KT990401 | China | Wu et al., 2016a |
| *Cy. brunneoruber* | HKAS80579-1 | – | KT990568 | KT990763 | – | – | China | Wu et al., 2016a |
| *Cy. instabilis* | HKAS59554 | – | KF112412 | KF112186 | – | – | China | Wu et al., 2014 |
| *Cy. pulverulentus* | 9606 | – | KF030313 | KF030418 | – | – | USA | Nuhn et al., 2013 |
| *Cy. sinopulverulentus* | HKAS59609 | – | KF112366 | KF112193 | KF112529 | KF112700 | China | Wu et al., 2014 |
| *ErythroPh. cinnabarinus* | GDGM46541 | – | MH374043 | MH378803 | MH374030 | MH374034 | China | Zhang and Li, 2018 |
| *Fistulinella prunicolor* | REH9502 | – | JX889648 | JX889690 | – | – | Australia | Halling et al., 2012b |
| *Gastroboletus* sp*.* | HKAS 57093 | – | KF112326 | – | – | KF112655 | China | Wu et al., 2014 |
| *Guyanaporus albipodus* | TH8848 | – | HQ161868 | – | HQ161837 | – | Guyana | Dentinger et al., 2010 |
| *Gymnogaster boletoides* | NY01194009 REH9455 | – | KT990572 | KT990768 | KT990928 | KT990406 | Australia | Wu et al., 2016a |
| *Gyrodon* sp. | HKAS57588 | – | KF112348 | KF112275 | KF112640 | KF112817 | China | Wu et al., 2014 |
| *Harrya atrogrisea* | HKAS50542 | – | KT990694 | KT990880 | KT991024 | KT990499 | China | Wu et al., 2016a |
| *Ha. chromapes* | HKAS59218 | – | HQ326932 | HQ326865 | – | – | China | Li et al., 2011 |
| *Heimioporus japonicus* | HKAS52237 | – | KF112347 | KF112228 | KF112618 | KF112806 | China | Wu et al., 2014 |
| *He. retisporus* | HKAS80583 | – | – | – | KT990929 | KT990408 | China | Wu et al., 2016a |
| *He. subretisporus* | HKAS80582 | – | KT990574 | KT990770 | – | KT990409 | China | Wu et al., 2016a |
| *Heliogaster columellifer* | KPM-NC 23012 | – | KX685724 | KX685718 | – | – | Africa | Orihara and Smith, 2017 |
| *Hemiaustroboletus vinaceobrunneus* | MEXU_30051 | – | MN200222 | – | – | – | Mexico | Ayala-Vásquez et al., 2022 |
| *Hem. vinaceobrunneus* | MEXU_30051 Holotype | – | MN200222 | – | – | MT887617 | Russia | Shilova et al., 2021 |
| *Hem. vinaceus* | AV524 Paratype | – | MN200225 | – | – | – | Mexico | Ayala-Vásquez et al., 2022 |
| *Hemileccinum impolitum* | HKAS84869 | – | KT990575 | KT990771 | KT990930 | KT990410 | Germany | Wu et al., 2016a |
| *Hemi. indecorum* | HKAS63126 | – | JN205457 | — | — | JN205455 | China | Zeng et al., 2012 |
| *Hemi. rugosum* | HKAS84355 | – | KT990578 | KT990774 | KT990931 | KT990413 | China | Wu et al., 2016a |
| *Hortiboletus* aff. *rubellus* | MB03-033 | – | KF030294 | KF030419 | KF030371 | – | USA | Nuhn et al., 2013 |
| *Ho. amygdalinus* | HKAS54166 | – | KT990581 | KT990777 | KT990933 | KT990416 | China | Wu et al., 2016a |
| *Ho. amygdalinus* | HKAS54242 | – | KT990580 | KT990776 | – | KT990415 | China | Wu et al., 2016a |
| *Ho. campestris* | MICH KUO | – | MK601740 | MK721094 | – | MK766302 | USA | Kuo and Ortiz-Santana, 2020 |
|  | 8240502 |  |  |  |  |  |  |  |
| *Ho. rubellus* | MICH KUO  6081002 | – | MK601741 | MK721095 | – | MK766303 | USA | Kuo and Ortiz-Santana, 2020 |
| *Ho. subpaludosus* | HKAS59608 | – | KF112371 | KF112185 | KF112551 | KF112696 | China | Wu et al., 2014 |
| *Ho. subpaludosus* | HKAS52659 | – | KT990582 | KT990778 | – | KT990417 | China | Wu et al., 2016a |
| *Ho. subpaludosus* | HKAS68158 | – | KT990583 | KT990779 | KT990934 | KT990418 | China | Wu et al., 2016a |
| *Hourangia cheoi* | HKAS52269 | – | KF112385 | KF112286 | KF112628 | KF112773 | China | Wu et al., 2014 |
| *Hou. cheoi* | HKAS74744 | – | KF112384 | KF112285 | KF112627 | KF112772 | China | Wu et al., 2014 |
| *Hou. cheoi* | Zhu130 KUN-HKAS 68306 | KP137001 | KP136950 | KP136929 | – | – | China | Zhu et al., 2015 |
| *Hou. cheoi* | Wu203 KUN-HKAS 59790 | KP136995 | KP136948 | KP136939 | – | – | China | Zhu et al., 2015 |
| *Hymenoboletus luteopurpureus* | HKAS46334 | – | KF112471 | KF112271 | KF112581 | KF112795 | China | Wu et al., 2014 |
| *Imleria badia* | HKAS74714 | – | KF112375 | – | KF112609 | – | China | Wu et al., 2014 |
| *Im. obscurebrunnea* | HKAS52557 | – | KF112374 | KF112190 | KF112608 | KF112707 | China | Wu et al., 2014 |
| *Im. pallida* | 179/97 | – | AF457409 | KF030424 | KF030396 | – | Germany | Nuhn et al., 2013 |
| *Indoporus shoreae* | AP 6693 | – | MK123973 | – | – | MK243367 | India | Parihar et al., 2018 |
| *In. shoreae* | AP6693 type | – | MK123973 | – | – | MK243367 | India | Parihar et al., 2018 |
| *In. shoreae* | AP6697 | – | MK123976 | – | – | MK243368 | India | Parihar et al., 2018 |
| *In. squamulosus* | HKAS76299 | – | MT154708 | MT110334 | MT110375 | – |  |  |
| *In. squamulosus* | HKAS107153 | – | MT154709 | MT110335 | MT110376 | MT110409 |  |  |
| *In. squamulosus* | HKAS84835 | – | MT154707 | – | – | – | China | Li and Yang, 2021 |
| *Ionosporus longipes* | Lee1180 | – | – | MT085471 | – | MH712031 | Australia | Khmelnitsky et al., 2019 |
| *Jimtrappea guyanensis* |  | – | LC053660 | – | LC053661 | – | Guyana | Smith et al., 2015 |
| *Kaziboletus rufescens* | HKAS74705 | – | JQ928620 | JQ928579 | JQ928589 | JQ928599 | Bangladesh | Hosen et al., 2013 |
| *Kombocles bakaiana* | THDJA 136 | – | KX827003 | – | – | – | Cameroon | Castellano et al., 2016 |
| ***Lanmaoa angustispora*** | **HMJAU 60054** | **–** | **OP373200** | **OP495820** | – | **OP495818** | **China** | **This study** |
| ***Lanmaoa angustispora*** | **HMJAU 60055** | **–** | **OP373201** | **OP495821** | – | **OP495819** | **China** | **This study** |
| ***Lanmaoa angustispora*** | **HMJAU 60056** | **–** | **–** | **OP495822** | **OP495823** | – | **China** | **This study** |
| *Lan. angustispora* | HKAS74752 | – | KM605139 | KM605154 | KM605166 | KM605177 | China | Wu et al., 2016b |
| *Lan. angustispora* | HKAS74765 | – | KF112322 | KF112159 | – | – | China | Wu et al., 2014 |
| *Lan. angustispora* | HKAS74752 | – | KM605139 | KM605154 | KM605166 | KM605177 | China | Wu et al., 2016b |
| *Lan. asiatica* | HKAS54094 | – | KF112353 | KF112161 | KF112522 | KF112682 | China | Wu et al., 2014 |
| *Lan. asiatica* | HKAS54094 | – | KF112353 | KF112161 | – | – | China | Wu et al., 2014 |
| *Lan. asiatica* | HKAS54095 | – | KM605141 | KM605151 | KM605164 | KM605174 | China | Wu et al., 2016b |
| *Lan. asiatica* | HKAS63516 | – | KT990584 | KT990780 | KT990935 | KT990419 | China | Wu et al., 2016a |
| *Lan. asiatica* | HKAS63592 | – | KM605142 | KM605152 | KM605163 | KM605175 | China | Wu et al., 2016b |
| *Lan. asiatica* | HKAS63603 | – | KM605143 | KM605153 | KM605165 | KM605176 | China | Wu et al., 2016b |
| *Lan. asiatica* | N K Zeng2125 | – | MG030470 | MG030481 | – | – | China | Chai et al., 2018 |
| *Lan. asiatica* | N K Zeng2795 | – | MG030469 | MG030480 | – | – | China | Chai et al., 2018 |
| *Lan. asiatica* | OR0228 | – | – | MH614730 | – | MH614777 | Thailand | Vadthanarat et al., 2019 |
| *Lan. borealis* | 2858 | – | JQ326998 | JQ327021 | – | – | USA | Halling et al., 2012a |
| *Lan. carminipes* | MB06 061 | – | JQ327001 | JQ327022 | KF030363 | – | USA | Nuhn et al., 2013 |
| *Lan. flavorubra* | NY775777 | – | JQ924339 | KF112160 | – | KF112681 | Costa Rica | Wu et al., 2014 |
| *Lan. macrocarpa* | N K Zeng3021 | – | – | MH879713 | – | – | China | Chai et al., 2019 |
| *Lan. macrocarpa* | N K Zeng3251 | – | MH879685 | MH885347 | – | – | China | Chai et al., 2019 |
| *Lan. pallidorosea* | BOTH4432 | – | – | MG897427 | – | MG897437 |  |  |
| *Lan. pallidorosea* | MO 210760 | – | MH216001 | MH318610 | – | – | USA |  |
| *Lan. pallidorosea* | MO 247881 | – | MH230088 | MH337278 | – | – | USA |  |
| *Lan. pseudosensibilis* | DS615 07 | – | KF030257 | KF030407 | – | – | USA | Nuhn et al., 2013 |
| *Lan. roseocrispans* | HOLOTYPE | – | MH036169 | KP327616 | – | – | USA | Bessette, AR. 2015. Nomenclatural novelties. Index Fungorum. 259:1-1 |
| *Lan. rubriceps* | FHMU 1756 | – | MG030465 | – | – | – | China | Chai et al., 2018 |
| *Lan. rubriceps* | FHMU 1757 | – | MG030467 | – | – | – | China | Chai et al., 2018 |
| *Lan. rubriceps* | FHMU 2801 | – | MG030471 | – | – | – | China | Chai et al., 2018 |
| *Lan. rubriceps* | N K Zeng2773 | – | MG030468 | MG030479 | – | – | China | Chai et al., 2018 |
| *Lan. rubriceps* | N K Zeng3006 | – | MH879683 | MH879712 | – | – | China | Chai et al., 2018 |
| *Lanmaoa* sp. | HKAS52518 | – | KF112354 | KF112162 | – | – | China | Wu et al., 2014 |
| *Lan. sublurida* | Farid 1023 | – | MW662572 | MW737485 | MW737498 | MW737460 | USA | Farid A et al., 2021 |
| *Lan. sublurida* | Farid 343 | – | MW662575 | MW737486 | MW737499 | MW737462 | USA | Farid A et al., 2021 |
| *Lan. sublurida* | Farid 631 | – | MW662578 | MW737487 | MW737501 | MW737464 | USA | Farid A et al., 2021 |
| *Lan. angustispora* | HKAS74759 | – | KM605140 | KM605155 | KM605167 | KM605178 | China | Wu et al., 2016b |
| *Leccinellum corsicum* | Buf4507 | – | KF030347 | KF030435 | KF030389 | – | USA | Nuhn et al., 2013 |
| *Le. crocipodium* | 930809/1 | – | AF139694 | – | – | – | France | Binder and Besl, 2000 |
| *Leccinellum* sp. | HKAS53427 | – | KF112488 | KF112253 | KF112596 | KF112727 | China | Wu et al., 2014 |
| *Leccinum quercinum* | HKAS63502 | – | KF112444 | KF112250 | KF112593 | KF112724 | China | Wu et al., 2014 |
| *Lec. scabrum* | HKAS56371 | – | KT990587 | KT990782 | – | KT990423 | China | Wu et al., 2016a |
| *Lec. variicolor* | HKAS57758 | – | KF112445 | KF112251 | KF112591 | KF112725 | China | Wu et al., 2014 |
| *Longistriata flava* | GT-2016a | – | – | LT574844 | – | – | Brazil | Sulzbacher et al., 2020 |
| *Mucilopilus castaneiceps* | HKAS71039 | – | KT990547 | KT990748 | KT990915 | KT990385 | China | Wu et al., 2016a |
| *Mu. castaneiceps* | HKAS50338 | – | KT990555 | KT990755 | KT990922 | KT990391 | China | Wu et al., 2016a |
| *Neotropicomus australis* | ACM1138 | – | OM068916 | – | – | – | Brazil | Magnago et al., 2022 |
| *Nigroboletus roseonigrescens* | ZT 13553 | – | KT220589 | KT220596 | KT220592 | KT220594 | China | Gelardi et al., 2015b |
| *Octaviania japonimontana* | KPM-C-0017812 | – | JN378486 | JN378428 | – | – | Japan | Orihara et al., 2012 |
| *Oc. tasmanica* | OSC132097 | – | JN378494 | JN378435 | – | – | Japan | Orihara et al., 2012 |
| *Parvixerocomus aokii* | HKAS59812 | – | KF112378 | KF112266 | KF112597 | — | China | Wu et al., 2014 |
| *Pa. pseudoaokii* | HKAS52633 | – | KF112379 | KF112267 | KF112598 | KF112736 | China | Wu et al., 2014 |
| *Paxillus obscurosporus* | Po1 | – | AY177256 | KF030442 | – | – | Germany | Nuhn et al., 2013 |
| *Phylloboletellus chloephorus* | 3388 | – | DQ534658 | – | – | – |  | Binder and Hibbett, 2006 |
| *Phylloporopsis boletinoides* | JBSD127411 | – | MH571711 | MH588312 | – | – | USA | Farid et al., 2018 |
| *Phylloporus alboinfuscatus* | Y.L. Chen DX010 FHMU5883 | MW588666 | MW588630 | – | – | – | China | Wu et al., 2021 |
| *Ph. alboinfuscatus* | N.K. Zeng4179 FHMU3276 | MW588663 | MW588626 | – | – | – | China | Wu et al., 2021 |
| *Ph. alboinfuscatus* | JXSB1620 | – | MK765818 | – | – | – |  |  |
| *Ph. alborufus* | MAN022 | JQ003624 | JQ003678 | – | – | – | Costa Rica | Neves et al., 2012 |
| *Ph. arenicola* | JT27954 | – | JQ003704 | – | – | – | USA | Neves et al., 2012 |
| *Ph. attenuatus* | KUN-HKAS 76168 | KR094777 | KR094781 | KR094791 | – | – | Bangladesh | Hosen and Li, 2017 |
| *Ph. attenuatus* | KUN-HKAS 76167 | KR094776 | KR094780 | KR094790 | – | – | Bangladesh | Hosen and Li, 2017 |
| *Ph. bellus* | OR0473 | – | – | MH580798 | – | – | China | Chuankid et al., 2019 |
| *Ph. bellus* | KUN-HKAS 56763 | JQ967239 | JQ967196 | JQ967153 | – | – | China | Zeng et al., 2013 |
| *Ph. bellus* | KUN-HKAS 42850 | JQ967240 | JQ967197 | JQ967154 | – | – | China | Zeng et al., 2013 |
| *Ph. bellus* | MCA559 | – | AY612817 | – | – | – | USA | Drehmel et al., 2008 |
| *Ph. bellus* | Y.G Fan2750 FHMU3285 | MW588655 | MW588589 | – | – | – | China | Wu et al., 2021 |
| ***Ph. biyangensis*** | **w3047 HMJAU60057** | **OP494681** | **–** | **OP495810** | **–** | **–** | **China** | **This study** |
| ***Ph. biyangensis*** | **w3048 HMJAU 60058** | **OP391534** | **OP377733** | **OP495811** | **–** | **–** | **China** | **This study** |
| ***Ph. biyangensis*** | **w3049 HMJAU 60059** | **OP391535** | **OP377734** | **–** | **–** | **–** | **China** | **This study** |
| ***Ph. biyangensis*** | **w3049b HMJAU 60060** | **OP391536** | **–** | **–** | **–** | **–** | **China** | **This study** |
| *Ph. bogoriensis* | DED7785 | JQ003625 | JQ003680 | – | – | – | Indonesia | Neves et al., 2012 |
| *Ph. bogoriensis* | N.K. Zeng761 FHMU459 | – | MW588592 | – | – | – | China | Wu et al., 2021 |
| *Ph. brunneiceps* | KUN-HKAS 56903 | JQ967241 | JQ967198 | JQ967155 | – | – | China | Zeng et al., 2013 |
| *Ph. brunneiceps* | KUN-HKAS 59551 | JQ967242 | JQ967199 | JQ967156 | – | – | China | Zeng et al., 2013 |
| *Ph. brunneiceps* | KUN-HKAS 59726 | JQ967243 | JQ967200 | JQ967157 | – | – | China | Zeng et al., 2013 |
| *Ph. brunneiceps* | KUN-HKAS 59727 | JQ967244 | JQ967201 | JQ967158 | – | – | China | Zeng et al., 2013 |
| *Ph. brunneiceps* | Y.J. Hao1427 FHMU3292 | – | MW588582 | MW574465 | – | – | China | Wu et al., 2021 |
| *Ph. brunneiceps* | JXSB1264 | – | MK765819 | – | – | – |  |  |
| *Ph. brunneiceps* | OR050 | – | – | KT824034 | – | – | Thailand | Raspé et al., 2016 |
| *Ph. caballeroi* | REH7906 | JQ003638 | JQ003662 | – | – | – | Panama | Neves et al., 2012 |
| *Ph. castanopsidis* | MAN104 | JQ003642 | JQ003689 | – | – | – | Thailand | Neves et al., 2012 |
| *Ph. castanopsidis* | MAN118 | JQ003646 | JQ003693 | – | – | – | Thailand | Neves et al., 2012 |
| *Ph. castanopsidis* | N.K. Zeng2278 FHMU1517 | MT822942 | MT829130 | – | – | – | China | Zeng and Jiang, 2020 |
| *Ph. catenulatus* | KUN-HKAS 76157 | KR094775 | KR094779 | KR094789 | – | – | Bangladesh | Hosen and Li, 2017 |
| *Ph. catenulatus* | KUN-HKAS 76156 | KR094774 | KR094778 | KR094788 | – | – | KR094788 | Hosen and Li, 2017 |
| *Ph. centroamericanus* | MAN016 | JQ003637 | JQ003663 | – | – | – | Costa Rica | Neves et al., 2012 |
| *Ph. centroamericanus* | MAN037 | JQ003634 | JQ003664 | – | – | – | Costa Rica | Neves et al., 2012 |
| *Ph. cyanescens* | REH8681 | JQ003621 | JQ003684 | – | – | – | Australia | Neves et al., 2012 |
| *Ph. dimorphus* | MAN128 | JQ003648 | JQ003697 | – | – | – | Thailand | Neves et al., 2012 |
| *Ph. foliiporus* | JLM1677 | JQ003641 | JQ003687 | – | – | – | USA | Neves et al., 2012 |
| *Ph. gajari* | KUN-HKAS 81585 | KP780419 | KP780423 | – | – | – | Bangladesh | Hosen and Li, 2015 |
| *Ph. gajari* | KUN-HKAS 76166 | KP780418 | KP780422 | – | – | – | Bangladesh | Hosen and Li, 2015 |
| *Ph. gajari* | KUN-HKAS 76161 | KP780417 | KP780421 | – | – | – | Bangladesh | Hosen and Li, 2015 |
| *Ph. grossus* | N.K. Zeng4159 FHMU3277 | MW588662 | MW588625 | – | – | – | China | Wu et al., 2021 |
| *Ph. grossus* | N.K. Zeng239 FHMU2469 | – | MW588583 | – | – | – | China | Wu et al., 2021 |
| *Ph. grossus* | N.K. Zeng4158 FHMU3280 | – | MW588624 | – | – | – | China | Wu et al., 2021 |
| *Ph. grossus* | N.K. Zeng3309 FHMU2270 | MW588642 | MW588586 | MW574462 | – | – | China | Wu et al., 2021 |
| *Ph. grossus* | N.K. Zeng3335 FHMU3136 | MW588641 | MW588585 | MW574464 | – | – | China | Wu et al., 2021 |
| *Ph. grossus* | N.K. Zeng3334 FHMU2937 | MW588640 | MW588584 | MW574463 | – | – | China | Wu et al., 2021 |
| *Ph. imbricatus* | HKAS54647 | – | JQ967202 | JQ967159 | — | — | China | Zeng et al., 2013 |
| *Ph. imbricatus* | KUN-HKAS 54647 | JQ967245 | JQ967202 | JQ967159 | – | – | China | Zeng et al., 2013 |
| *Ph. imbricatus* | KUN-HKAS 54859 | JQ967246 | JQ967203 | JQ967160 | – | – | China | Zeng et al., 2013 |
| *Ph. imbricatus* | KUN-HKAS 54860 | JQ967247 | JQ967204 | JQ967161 | – | – | China | Zeng et al., 2013 |
| *Ph. imbricatus* | KUN-HKAS 54861 | JQ967248 | JQ967205 | JQ967162 | – | – | China | Zeng et al., 2013 |
| *Ph. imbricatus* | KUN-HKAS 68642 | – | KF112398 | KF112299 | – | – | China | Wu et al., 2014 |
| *Ph. infuscatus* | MAN123 | – | JQ003695 | – | – | – | Thailand | Neves et al., 2012 |
| *Ph. leucomycelinus* | MB00-043 | JQ003628 | JQ003677 | – | – | – | USA | Neves et al., 2012 |
| *Ph. leucomycelinus* | KUN-HKAS 74678 | JQ967249 | JQ967206 | JQ967163 | – | – | China | Zeng et al., 2013 |
| *Ph. luxiensis* | N.K. Zeng3439 FHMU3095 | – | – | MW574467 | – | – | China | Wu et al., 2021 |
| *Ph. luxiensis* | N.K. Zeng3442 FHMU3101 | – | MW588605 | MW574469 | – | – | China | Wu et al., 2021 |
| *Ph. luxiensis* | N.K. Zeng3642 FHMU2996 | – | MW588609 | MW574466 | – | – | China | Wu et al., 2021 |
| *Ph. luxiensis* | N.K. Zeng2784 FHMU1773 | – | MW588608 | – | – | – | China | Wu et al., 2021 |
| *Ph. luxiensis* | N.K. Zeng4196 FHMU3281 | – | MW588628 | – | – | – | China | Wu et al., 2021 |
| *Ph. luxiensis* | N.K. Zeng2782 FHMU1771 | – | MW588610 | – | – | – | China | Wu et al., 2021 |
| *Ph. luxiensis* | N.K. Zeng1364 FHMU915 | MW588643 | MW588607 | – | – | – | China | Wu et al., 2021 |
| *Ph. luxiensis* | N.K. Zeng2789 FHMU2935 | – | MW588606 | – | – | – | China | Wu et al., 2021 |
| *Ph. luxiensis* | N.K. Zeng3444 FHMU3105 | MW588644 | MW588611 | MW574468 | – | – | China | Wu et al., 2021 |
| *Ph. luxiensis* | KUN-HKAS 57036 | JQ967250 | JQ967207 | JQ967164 | – | – | China | Zeng et al., 2013 |
| *Ph. luxiensis* | KUN-HKAS 57048 | JQ967252 | JQ967209 | JQ967166 | – | – | China | Zeng et al., 2013 |
| *Ph. luxiensis* | KUN-HKAS 75077 | – | KF112490 | KF112298 | – | – | China | Wu et al., 2014 |
| *Ph. luxiensis* | KUN-HKAS 74680 | JQ967272 | JQ967229 | JQ967185 | – | – | China | Zeng et al., 2013 |
| *Ph. luxiensis* | KUN-HKAS 74684 | JQ967275 | JQ967232 | JQ967188 | – | – | China | Zeng et al., 2013 |
| *Ph. maculatus* | KUN-HKAS 56683 | JQ967253 | JQ967210 | JQ967167 | – | – | China | Zeng et al., 2013 |
| *Ph. maculatus* | KUN-HKAS 59730 | JQ678696 | JQ678698 | JQ967194 | – | – | China | Zeng et al., 2013 |
| *Ph. maculatus* | OR0285 | – | – | MH580800 | – | – | China | Chuankid et al., 2019 |
| *Ph. maculatus* | Y.G. Fan2752 FHMU3287 | MW588656 | MW588618 | – | – | – | China | Wu et al., 2021 |
| *Ph. microsquamus* | OR0258 | – | – | MH580809 | – | – | China | Wu et al., 2021 |
| *Ph. microsquamus* | N.K. Zeng2571 FHMU1678 | MW588648 | MW588599 | – | – | – | China | Wu et al., 2021 |
| *Ph. microsquamus* | N.K. Zeng189 FHMU2467 | – | MW588594 | – | – | – | China | Wu et al., 2021 |
| *Ph. microsquamus* | N.K. Zeng1990 FHMU1285 | MW588649 | MW588597 | MW574472 | – | – | China | Wu et al., 2021 |
| *Ph. microsquamus* | N.K. Zeng2523 FHMU1640 | MW588647 | MW588598 | – | – | – | China | Wu et al., 2021 |
| *Ph. microsquamus* | N.K. Zeng3549 FHMU3012 | MW588653 | MW588613 | MW574473 | – | – | China | Wu et al., 2021 |
| *Ph. microsquamus* | N.K. Zeng3560 FHMU3300 | MW588654 | MW588614 | MW574474 | – | – | China | Wu et al., 2021 |
| *Ph. microsquamus* | N.K. Zeng3556 FHMU3018 | MW588650 | MW588600 | MW574471 | – | – | China | Wu et al., 2021 |
| *Ph. microsquamus* | N.K. Zeng2154 FHMU1416 | MW588651 | MW588595 | – | – | – | China | Wu et al., 2021 |
| *Ph. microsquamus* | N.K. Zeng3551 FHMU3064 | MW588652 | MW588596 | MW574470 | – | – | China | Wu et al., 2021 |
| *Ph. nigrisquamus* | Y.G. Fan2819 FHMU3271 | – | MW588590 | – | – | – | China | Wu et al., 2021 |
| *Ph. nigrisquamus* | Y.G. Fan2834 FHMU3272 | – | MW588591 | – | – | – | China | Wu et al., 2021 |
| *Ph. nigrisquamus* | KUN-HKAS 63001 | – | KF112396 | – | – | – | China | Wu et al., 2014 |
| *Ph. nigrisquamus* | MAN131 | JQ003649 | JQ003698 | – | – | – | Thailand | Neves et al., 2012 |
| *Ph. nigrobrunneus* | KUN-HKAS 74683 | JQ967274 | JQ967231 | JQ967187 | – | – | China | Zeng et al., 2013 |
| *Ph. nigrobrunneus* | KUN-HKAS 74682 | JQ967273 | JQ967230 | JQ967186 | – | – | China | Zeng et al., 2013 |
| *Ph. nigrobrunneus* | REH4582 | – | JQ003679 | – | – | – | USA | Neves et al., 2012 |
| *Ph. nigrobrunneus* | OR0448 | – | – | MG212598 | – | – | Thailand | Vadthanarat et al., 2018 |
| *Ph. nigrobrunneus* | Y.G. Fan2753 FHMU3269 | MW588637 | MW588603 | – | – | – | China | Wu et al., 2021 |
| *Ph. nigrobrunneus* | Y.G. Fan2754 FHMU3268 | MW588638 | MW588604 | – | – | – | China | Wu et al., 2021 |
| *Ph. nigrobrunneus* | Y.G. Fan2749 FHMU3270 | MW588636 | MW588602 | – | – | – | China | Wu et al., 2021 |
| *Ph. orientalis* | REH8755 | JQ003651 | JQ003701 | – | – | – | Australia | Neves et al., 2012 |
| *Ph. orientalis* | REH8756 | JQ003652 | JQ003709 | – | – | – | Australia | Neves et al., 2012 |
| *Ph. pachycystidiatus* | KUN-HKAS 54540 | JQ967254 | JQ967211 | JQ967168 | – | – | China | Zeng et al., 2013 |
| *Ph. pachycystidiatus* | KUN-HKAS 54541 | JQ967255 | JQ967212 | JQ967169 | – | – | China | Zeng et al., 2013 |
| *Ph. pachycystidiatus* | N.K. Zeng3622 FHMU2834 | MW588658 | MW588616 | MW574459 | – | – | China | Wu et al., 2021 |
| *Ph. pachycystidiatus* | N.K. Zeng3553 FHMU3035 | MW588659 | MW588617 | MW574458 | – | – | China | Wu et al., 2021 |
| *Ph. pachycystidiatus* | N.K. Zeng3607 FHMU3023 | MW588660 | MW588615 | MW574457 | – | – | China | Wu et al., 2021 |
| *Ph. parvisporus* | KUN-HKAS 54768 | JQ967257 | JQ967214 | JQ967171 | – | – | China | Zeng et al., 2013 |
| *Ph. parvisporus* | KUN-HKAS 59725 | JQ967256 | JQ967213 | JQ967170 | – | – | China | Zeng et al., 2013 |
| *Ph. pelletieri* | K 128205 | JQ967258 | JQ967215 | – | – | – | China | Zeng et al., 2013 |
| *Ph. pelletieri* | Q7199c | JQ003639 | JQ003668 | – | – | – | Slovakia | Neves et al., 2012 |
| *Ph. phaeoxanthus* | MAN064 | – | JQ003670 | – | – | – | Costa Rica | Neves et al., 2012 |
| *Ph. phaeoxanthus var. simplex* | REH7388 | – | JQ003671 | – | – | – | Costa Rica | Neves et al., 2012 |
| *Ph. pruinatus* | KUN-HKAS 74687 | JQ967278 | JQ967235 | JQ967190 | – | – | China | Zeng et al., 2013 |
| *Ph. pruinatus* | KUN-HKAS 101929 | MH624124 | MH624125 | MH624126 | – | – | China |  |
| *Ph. purpurellus* | MAN050 | JQ003630 | JQ003672 | – | – | – | Costa Rica | Neves et al., 2012 |
| *Ph. pusillus* | OR0484 | – | – | MH580802 | – | – | China | Chuankid et al., 2019 |
| *Ph. pusillus* | OR1158 | – | – | MH580803 | – | – | Thailand | Chuankid et al., 2019 |
| *Ph. pusillus* | OR1310 | – | – | MH580804 | – | – | Thailand | Chuankid et al., 2019 |
| *Ph. quercophilus* | Montoya_5239 | MK226550 | MK226558 | – | – | – | Mexico | Montoya et al., 2019 |
| *Ph. rhodoxanthus* | MAN075 | – | JQ003674 | – | – | – | USA | Neves et al., 2012 |
| *Ph. rhodoxanthus* | REH8714 | JQ003629 | JQ003675 | – | – | – | USA | Neves et al., 2012 |
| *Ph. rhodoxanthus* | MAN099 | – | JQ003676 | – | – | – | USA | Neves et al., 2012 |
| *Ph. rhodoxanthus* | JLM1808 | JQ003654 | JQ003688 | – | – | – | USA | Neves et al., 2012 |
| *Ph. rimosus* | Montoya_4834 | MK226546 | MK226554 | MK314102 | – | – | Mexico | Montoya et al., 2019 |
| *Ph. rubeolus* | HKAS52573 | JQ967259 | JQ967216 | JQ967172 | — | — | China | Zeng et al., 2013 |
| *Ph. rubeolus* | KUN-HKAS 54543 | JQ967261 | JQ967218 | JQ967174 | – | – | China | Zeng et al., 2013 |
| *Ph. rubeolus* | OR0251 | – | – | MH580806 | – | – | China | Chuankid et al., 2019 |
| *Ph. rubeolus* | N.K. Zeng2459 FHMU1585 | MT822943 | MT829131 | – | – | – | China | Zeng and Jiang, 2020 |
| *Ph. rubeolus* | N.K. Zeng3614 FHMU3301 | MW588639 | MW588619 | MW574455 | – | – | China | Wu et al., 2021 |
| *Ph. rubiginosus* | MAN119 | JQ003647 | JQ003694 | – | – | – | Thailand | Neves et al., 2012 |
| *Ph. rubiginosus* | MAN117 | JQ003645 | JQ003692 | – | – | – | Thailand | Neves et al., 2012 |
| *Ph. rubiginosus* | BC063 | – | – | MH580807 | – | – | Thailand | Chuankid et al., 2019 |
| *Ph. rubiginosus* | OR0169 | – | – | MH580808 | – | – | Thailand | Chuankid et al., 2019 |
| *Ph. rubiginosus* | N.K. Zeng589 FHMU2482 | MK116512 | MK116509 | – | – | – | China | Zhang et al., 2019 |
| *Ph. rubiginosus* | N.K. Zeng3175 FHMU2136 | – | MK116511 | – | – | – | China | Zhang et al., 2019 |
| *Ph. rubiginosus* | N.K. Zeng3055 FHMU2016 | MT822964 | MT829149 | MW574454 | – | – | China | Wu et al., 2021 |
| *Ph. rubrosquamosus* | KUN-HKAS 54542 | JQ967260 | JQ967217 | JQ967173 | – | – | China | Zeng et al., 2013 |
| *Ph. rubrosquamosus* | KUN-HKAS 54559 | JQ967262 | JQ967219 | JQ967175 | – | – | China | Zeng et al., 2013 |
| *Ph. rubrosquamosus* | KUN-HKAS 52552 | – | KF112391 | KF112289 | – | – | China | Wu et al., 2014 |
| *Ph. rufescens* | KUN-HKAS 59722 | JQ967263 | JQ967220 | JQ967176 | – | – | China | Zeng et al., 2013 |
| *Ph. rufescens* | KUN-HKAS 59723 | JQ967264 | JQ967221 | JQ967177 | – | – | China | Zeng et al., 2013 |
| *Ph. rufescens* | N.K. Zeng1904 FHMU1227 | MW588645 | – | MW574453 | – | – | China | Wu et al., 2021 |
| *Phylloporus* sp. | REH7733 | – | JQ003661 | – | – | – | Costa Rica | Neves et al., 2012 |
| *Phylloporus* sp. | REH8710 | JQ003618 | JQ003686 | – | – | – | USA | Neves et al., 2012 |
| *Phylloporus* sp. | N.K. Zeng3445 FHMU3283 | MW588634 | – | MW574461 | – | – | China | Wu et al., 2021 |
| *Phylloporus* sp. | N.K. Zeng4088 FHMU3363 | – | MW588621 | – | – | – | China | Wu et al., 2021 |
| *Phylloporus* sp. | N.K. Zeng2724 FHMU1718 | MW588632 | MW588581 | – | – | – | China | Wu et al., 2021 |
| *Phylloporus* sp. | N.K. Zeng3558 FHMU3362 | – | – | MW574456 | – | – | China | Wu et al., 2021 |
| *Phylloporus* sp. | N.K. Zeng4228 FHMU3282 | MW588665 | MW588629 | – | – | – | China | Wu et al., 2021 |
| *Phylloporus* sp. | JXSB1345 | – | MK765820 | – | – | – |  |  |
| *Phylloporus* sp. | OR052 | – | – | KT824035 | – | – | Thailand | Raspé et al., 2016 |
| *Phylloporus* sp. | BC074 | – | – | MH580799 | – | – | Thailand | Chuankid et al., 2019 |
| *Phylloporus* sp. | WU17978 | – | – | MH580805 | – | – | Austria | Chuankid et al., 2019 |
| *Phylloporus* sp. | SAR 89.457 | – | U11925 | – | – | – | USA | Chapela et al., 1994 |
| *Phylloporus* sp. | REH8729 | JQ003650 | JQ003699 | – | – | – | Australia | Neves et al., 2012 |
| *Phylloporus* sp. | MAN105 | – | JQ003690 | – | – | – | Thailand | Neves et al., 2012 |
| *Phylloporus* sp. | KUN-HKAS 74679 | JQ967271 | JQ967228 | JQ967184 | – | – | China | Zeng et al., 2013 |
| *Phylloporus* sp. | KUN-HKAS 74681 | JQ967270 | JQ967227 | JQ967183 | – | – | China | Zeng et al., 2013 |
| *Phylloporus* sp. | KUN-HKAS 74686 | JQ967277 | JQ967234 | – | – | – | China | Zeng et al., 2013 |
| *Phylloporus* sp. | KUN-HKAS 74685 | JQ967276 | JQ967233 | JQ967189 | – | – | China | Zeng et al., 2013 |
| *Ph. subbacillisporus* | OR0989 | MH686277 | – | – | – | – |  | Chuankid et al., 2019 |
| *Ph. subbacillisporus* | Y.J. Hao1553 FHMU3293 | MW588635 | MW588601 | MW574460 | – | – | China | Wu et al., 2021 |
| *Ph. subrubeolus* | BC022 | – | – | MH580813 | – | – | Thailand | Chuankid et al., 2019 |
| *Ph. subrubeolus* | OR0612 | – | – | MH580814 | – | – | Thailand | Chuankid et al., 2019 |
| *Ph. subrubeolus* | N.K. Zeng4027 FHMU3275 | – | MW588620 | MW574475 | – | – | China | Wu et al., 2021 |
| *Ph. subrubeolus* | N.K. Zeng4195 FHMU3273 | MW588664 | MW588627 | MW574476 | – | – | China | Wu et al., 2021 |
| *Ph. subrubeolus* | N.K. Zeng4089 FHMU3278 | – | MW588622 | – | – | – | China | Wu et al., 2021 |
| *Ph. subrubeolus* | N.K. Zeng4095 FHMU3274 | MW588661 | MW588623 | – | – | – | China | Wu et al., 2021 |
| *Ph. subrubeolus* | KUN-HKAS 74688 | JQ967279 | JQ967236 | JQ967191 | – | – | China | Zeng et al., 2013 |
| *Ph. tenuissimus* | KUN-HKAS 74689 | JQ967280 | JQ967237 | JQ967192 | – | – | China | Zeng et al., 2013 |
| *Ph. tenuissimus* | N.K. Zeng3514 FHMU2964 | MW588646 | MW588593 | MW574452 | – | – | China | Wu et al., 2021 |
| *Ph. yunnanensis* | KUN-HKAS 52225 | JQ967265 | JQ967222 | JQ967178 | – | – | China | Zeng et al., 2013 |
| *Ph. yunnanensis* | KUN-HKAS 52527 | JQ967266 | JQ967223 | JQ967179 | – | – | China | Zeng et al., 2013 |
| *Ph. yunnanensis* | KUN-HKAS 56999 | JQ967267 | JQ967224 | JQ967180 | – | – | China | Zeng et al., 2013 |
| *Ph. yunnanensis* | KUN-HKAS 58673 | JQ967268 | JQ967225 | JQ967181 | – | – | China | Zeng et al., 2013 |
| ***Porphyrellus pseudocyaneotinctus*** | **w3019 HMJAU 60061** | **–** | **–** | **OP495802** | **OP495794** | **OP495786** | **China** | **this study** |
| ***Po. pseudocyaneotinctus*** | **w3039 HMJAU 60062** | **–** | **–** | **OP495803** | **OP495795** | **OP495787** | **China** | **this study** |
| ***Po. pseudocyaneotinctus*** | **w3046 HMJAU 60063** | **–** | **–** | **OP495804** | **OP495796** | **OP495788** | **China** | **this study** |
| ***Po. pseudocyaneotinctus*** | **w3054 HMJAU 60064** | **–** | **–** | **OP495805** | **OP495797** | **OP495789** | **China** | **this study** |
| ***Po. pseudocyaneotinctus*** | **w3062 HMJAU 60065** | **–** | **–** | **OP495806** | **OP495798** | **OP495790** | **China** | **this study** |
| ***Po. pseudocyaneotinctus*** | **w3085 HMJAU 60066** | **–** | **OP380692** | **OP495807** | **OP495799** | **OP495791** | **China** | **this study** |
| ***Po. pseudocyaneotinctus*** | **w3088 HMJAU 60067** | **–** | **OP380693** | **OP495808** | **OP495800** | **OP495792** | **China** | **this study** |
| ***Po. pseudocyaneotinctus*** | **w3091 HMJAU 60068** | **–** | **OP380694** | **OP495809** | **OP495801** | **OP495793** | **China** | **this study** |
| *Po. brunneus* | REH9508 | – | JX889646 | JX889688 | – | – | Australia | Halling et al., 2012b |
| *Po. castaneus* | HKAS52554 | – | KT990697 | KT990883 | KT991026 | KT990502 | China | Wu et al., 2016a |
| *Po. castaneus* | HKAS63076 | – | KT990548 | KT990749 | KT990916 | KT990386 | China | Wu et al., 2016a |
| *Po. castaneus* | HKAS52554 type | – | KT990697 | KT990883 | KT991026 | KT990502 | China | Wu et al., 2016a |
| *Po. cyaneotinctus* | HKAS80183 | – | MT154718 | MT110340 | – | – |  |  |
| *Po. cyaneotinctus* | HKAS80192 | – | MT154719 | – | – | – | China | Li and Yang, 2021 |
| *Po. griseus* | HKAS82849 type | – | MT154716 | – | – | MT110414 | China | Li and Yang, 2021 |
| *Po. nigropurpureus* | HKAS52685 | – | KT990627 | KT990821 | KT990973 | KT990459 | China | Wu et al., 2016a |
| *Po. orientifumosipes* | HKAS53372 type | – | KT990629 | KT990823 | KT990975 | KT990461 | China | Wu et al., 2016a |
| *Po. orientifumosipes* | HKAS84710 | – | MT154717 | MT110339 | – | MT110415 | China | Li and Yang, 2021 |
| *Po. porphyrosporus* | HKAS76671 | – | KF112482 | KF112243 | KF112611 | KF112718 | China | Wu et al., 2014 |
| *Po. porphyrosporus* | HKAS49182 | – | KT990544 | KT990746 | KT990912 | KT990383 | China | Wu et al., 2016a |
| *Po. pseudofumosipes* | HKAS103784 type | – | MW114845 | – | – | – | China | Li and Yang, 2021 |
| *Po. scrobiculatus* | HKAS53366 type | – | KF112480 | KF112241 | KF112610 | KF112716 | China | Wu et al., 2014 |
| *Pseudoaustroboletus valens* | HKAS52603 | – | KM274869 | KM274877 | – | – | China | Li et al., 2014a |
| *Ps. valens* | HKAS82644 | – | – | MT110359 | MT110396 | MT110431 | China | Li and Yang, 2021 |
| *Pseudoboletus parasiticus* | Xpa1 | – | AF050646 | KF030443 | KF030394 | – | Germany | Nuhn et al., 2013 |
| *Pulchroboletus roseoalbidus* | AMB12757 | – | KJ729499 | KJ729512 | — | — | Italy | Gelardi et al., 2014 |
| *Pulveroboletus mirus* | HKAS57628 | – | KT990618 | KT990812 | – | KT990453 | China | Wu et al., 2016a |
| *Pulveroboletus* sp. | HKAS55369 | – | KT990620 | KT990814 | – | KT990455 | China | Wu et al., 2016a |
| *Retiboletus brunneolus* | HKAS52680 | – | KF112424 | KF112179 | – | KF112690 | China | Wu et al., 2014 |
| *Re. fuscus* | HKAS63590 | – | KF112417 | KF112178 | KF112537 | KF112691 | China | Wu et al., 2014 |
| *Rheubarbariboletus persicolor* | ML41842RP | MH011927 | – | – | – | – |  | Loizides et al., 2019 |
| *Rhodactina rostratispora* |  | – | – | MG212606 | – | MG212646 | Thailand | Vadthanarat et al., 2018 |
| *Rossbeevera vittatispora* | OSC61484 | – | JN378506 | JN378446 | – | – | Australia | Orihara et al., 2012 |
| *Ro. yunnanensis* | HKAS70601 | – | KF112448 | – | – | KF112729 | China | Wu et al., 2014 |
| *Royoungia boletoides* | Trappe27456 | – | JX889655 | JX889696 | – | – | Australia | Halling et al., 2012b |
| *Roy. coccineinana* | HKAS68927 | – | KT990508 | – | KT990889 | KT990347 | China | Wu et al., 2016a |
| *Roy. palumanus* | REH9421 | – | JX889675 | JX889685 | – | – | Australia | Halling et al., 2012b |
| *Rubroboletus latisporus* | HKAS80358 | – | KP055023 | KP055020 | KP055026 | KP055029 | China | Zhao et al., 2014b |
| *Ru. sinicus* | HKAS68620 | – | KF112319 | KF112146 | KF112504 | KF112661 | China | Wu et al., 2014 |
| *Rugiboletus brunneiporus* | HKAS56359 | – | KF112401 | KF112196 | – | – | China | Wu et al., 2014 |
| *Rug. extremiorientalis* | HKAS74754 | – | KT990639 | KT990832 | KT990982 | KT990469 | China | Wu et al., 2016a |
| *Rugiboletus* sp. | HKAS55373 | – | KF112362 | KF112303 | KF112588 | KF112804 | China | Wu et al., 2016a |
| *Singerocomus rubriflavus* | Henkel 9585 | – | LC043093 | – | LC043094 | – | Guyana | Henkel et al., 2016 |
| *Solioccasus polychromus* | REH9417 | – | JQ287642 | JQ287644 | – | – | Australia | Trappe et al., 2013 |
| *Spongiforma thailandica* | DED7873 | – | EU685108 | KF030436 | KF030387 | – | Thailand | Nuhn et al., 2013 |
| *Sp. temasekensis* | SING 0206334 | – | MG979397 | MG979392 | MG979393 | – | Singapore | Wu et al., 2018 |
| *Strobilomyces atrosquamosus* | HKAS55368 | – | KT990648 | KT990839 | KT990989 | KT990476 | China | Wu et al., 2016a |
| *St. seminudus* | HKAS59461 | – | KF112479 | KF112260 | KF112606 | KF112815 | China | Wu et al., 2014 |
| *Suillellus amygdalinus* | NY00035656 Thiers54483 | – | KT990650 | KT990840 | KT990990 | KT990477 | USA | Wu et al., 2016a |
| *Su. subamygdalinus* | HKAS57953 | – | KT990652 | KT990842 | KT990992 | – | China | Wu et al., 2016a |
| *Sutorius alpinus* | HKAS50420 | – | KT990549 | KT990750 | KT990917 | KT990387 | China | Wu et al., 2016a |
| *Sut. alpinus* | HKAS59657 | – | KT990707 | KT990887 | KT991029 | KT990505 | China | Wu et al., 2016a |
| *Sut. brunneissimus* | HKAS 57451 | – | KM605137 | KM605149 | KM605161 | KM605172 | China | Wu et al., 2016b |
| *Sut. brunneissimus* | HKAS 50538 | – | KM605138 | KM605150 | KM605162 | KM605173 | China | Wu et al., 2016b |
| *Sut. eximius* | HKAS91261 | – | MT154770 | – | – | MT110444 | China | Li and Yang, 2021 |
| *Sut. hainanensis* | HKAS59469 | – | KF112359 | KF112175 | KF112500 | KF112669 | China | Zhu et al., 2015 |
| *Sut. microsporus* | HKAS56291 | – | KF112400 | KF112208 | KF112585 | KF112803 | China | Wu et al., 2014 |
| *Sut. rubriporus* | HKAS89181 | – | KT990603 | KT990797 | KT990952 | – | China | Wu et al., 2016a |
| *Sut. sanguineus* | HKAS80849 | – | KT990609 | KT990803 | KT990958 | KT990443 | China | Wu et al., 2016a |
| *Tengioboletus glutinosus* | HKAS53425 | – | KF112341 | KF112204 | KF112578 | KF112800 | China | Wu et al., 2014 |
| *Te. reticulatus* | HKAS53426 | – | KF112491 | KF112313 | KF112649 | KF112828 | China | Wu et al., 2014 |
| *Tubosaeta* sp*.* | 18543 | JF908794 | – | – | – | – |  |  |
| *Turmalinea yuwanensis* | KPM-NC-0018011 | – | KC552046 | KC552089 | – | – | Japan | Orihara et al., 2016 |
| *Tylocinum griseolum* | HKAS52612 | – | KT990631 | KT990825 | – | – | China | Wu et al., 2016a |
| *Ty. griseolum* | HKAS50281 | – | KF112451 | KF112284 | – | KF112730 | China | Wu et al., 2014 |
| *Tylopilus* aff. *chromapes* | 01-513 | – | JX889672 | JX889682 | – | – | Zambia | Halling et al., 2012b |
| *Tyl.* aff. *virens* | 01-541 | – | JX889677 | JX889687 | – | – | Zambia | Halling et al., 2012b |
| *Tylopilus atripurpureus* | HKAS50279 | – | HQ326935 | HQ326868 | – | – | China | Li et al., 2011 |
| *Tyl. atroviolaceobrunneus* | HKAS84351 | – | KT990625 | KT990819 | – | – | China | Wu et al., 2016a |
| *Tyl. otsuensis* | HKAS50240 | – | KT990553 | KT990753 | KT990921 | – | China | Wu et al., 2016a |
| *Tylopilus* sp. | HKAS50211 | – | KT990552 | KT990752 | KT990920 | KT990389 | China | Wu et al., 2016a |
| *Tylopilus* sp. | HKAS90198 | – | KT990559 | – | – | – | China | Wu et al., 2016a |
| *Tylopilus* sp. | HKAS50229 | – | KF112423 | KF112216 | KF112574 | KF112769 | China | Wu et al., 2014 |
| *Tylopilus* sp. | HKAS53367 | – | KF112439 | KF112304 | KF112615 | KF112790 | China | Wu et al., 2014 |
| *Tyl. violaceobrunneus* | HKAS89443 | – | KT990702 | KT990886 | KT991028 | KT990504 | China | Wu et al., 2016a |
| *Veloboletus limbatus* | REH9228 | – | – | MN413636 | – | MT747397 | Australia | Crous et al., 2020 |
| *Veloporphyrellus gracilioides* | HKAS53590 | – | KF112381 | KF112210 | KF112556 | KF112734 | China | Wu et al., 2014 |
| *Ve. pantoleucus* | Gomez21232 2 | – | JX984547 | – | – | – | Costa Rica | Li et al., 2014b |
| *Ve. pseudovelatus* | HKAS52258 | – | JX984540 | JX984551 | – | – | China | Li et al., 2014b |
| *Ve. velatus* | HKAS63668 | – | JX984546 | JX984554 | – | – | China | Li et al., 2014b |
| *Xanthoconium affine* | NY00815399 REH8660 | – | KT990661 | KT990850 | KT990999 | KT990486 |  | Wu et al., 2016a |
| *Xa. porophyllum* | HKAS90217 | – | KT990662 | KT990851 | KT991000 | KT990487 | China | Wu et al., 2016a |
| *Xerocomellus chrysenteron* | HKAS56494 | – | KF112357 | KF112172 | KF112526 | KF112685 | China | Wu et al., 2014 |
| *Xe. cisalpinus* | PDD94421 | – | JQ924322 | KF112171 | KF112525 | KF112686 | New Zealand | Wu et al., 2014 |
| *Xe. communis* | HKAS50467 | – | KT990670 | KT990858 | KT991008 | KT990494 | China | Wu et al., 2016a |
| *Xe. communis* | HKAS68204 | – | KT990671 | KT990859 | KT991009 | KT990495 | China | Wu et al., 2016a |
| *Xe. corneri* | HKAS52503 | – | KT990668 | KT990856 | KT991006 | KT990492 | China | Wu et al., 2016a |
| *Xe. corneri* | HKAS90206 | – | KT990669 | KT990857 | KT991007 | KT990493 | China | Wu et al., 2016a |
| *Xerocomus fulvipes* | HKAS52556 | – | KT990672 | KT990860 | KT991010 | – | China | Wu et al., 2016a |
| *Xer. subparvus* | HKAS53387 | – | KF112397 | KF112297 | KF112634 | KF112788 | China | Wu et al., 2014 |
| *Xer. yunnanensis* | HKAS68420 | – | KT990690 | KT990877 | KT991021 | – | China | Wu et al., 2014 |
| *Zangia erythrocephala* | HKAS52844 | – | HQ326944 | – | – | – | China | Li et al., 2011 |
| *Za. olivaceobrunnea* | HKAS52275 | – | HQ326947 | HQ326875 | – | – | China | Li et al., 2011 |
| *Za. roseola* | HKAS75046 | – | KF112414 | KF112269 | KF112579 | KF112791 | China | Wu et al., 2014 |
